# Soil-plant-animal properties and microbial reassembly distinguish cattle oral disease prevalence across converted Amazon pasture systems

**DOI:** 10.1007/s10653-026-03516-x

**Published:** 2026-10-07

**Authors:** Fernando Igne Rocha, Ana Carolina Borsanelli, Aline Pacobahyba de Oliveira, Cecílio Viega Soares Filho, Marcia Reed Rodrigues Coelho, Stefan Schwab, Carlos Magno dos Santos, Wenceslau Geraldes Teixeira, James Cole, Adina Howe, Iveraldo Santos Dutra, Ederson da Conceição Jesus

**Affiliations:** 1 Department of Soil, Federal Rural University of Rio de Janeiro, Seropédica, RJ Brazil; 2 Center for Nuclear Energy in Agriculture, University of São Paulo, Piracicaba, SP Brazil; 3 Department of Veterinary Medicine, Federal University of Goiás, Goiânia, GO Brazil; 4 National Soil Research Center, Embrapa Solos, Rio de Janeiro, RJ Brazil; 5 Department of Production and Animal Health, São Paulo State University Júlio de Mesquita Filho, Araçatuba, SP Brazil; 6 National Agrobiology Research Center, Embrapa Agrobiologia, Seropédica, RJ Brazil; 7 SENAI Innovation Institute for Green Chemistry, Rio de Janeiro, RJ Brazil; 8 Department of Plant, Soil and Microbial Sciences, Michigan State University, East Lansing, MI USA; 9 Department of Agricultural and Biosystems Engineering, Iowa State University, Ames, IA USA

**Keywords:** Amazon deforestation, Bovine periodontitis, Soil–plant-animal continuum, Microbial reassembly, Soil properties, One Health

## Abstract

Forest-to-pasture conversion can alter soil properties, forage composition, and microbial communities, but whether these changes are associated with bovine periodontitis remains unclear. We compared eight pasture systems in the Western Brazilian Amazon classified by high or low herd-level prevalence of periodontal lesions. Soil physicochemical properties, forage composition, microbial communities in soil, forage, and bovine subgingival biofilms, and the relative abundance of the streptomycin biosynthesis gene strB1 were evaluated. Although the systems had similar deforestation histories, low-prevalence pastures had finer-textured soils, greater soil Cu and forage Zn, and greater representation of Bacilli and Gammaproteobacteria. High-prevalence pastures had sandier soils, greater soil C:N ratios, lower micronutrient availability, and greater representation of Actinobacteria and Bacteroidetes. Microbial communities differed between prevalence classes in all three compartments, with higher alpha and gamma diversity and more compartmentalized co-occurrence networks in high-prevalence systems. Tax4Fun predicted greater representation of pathways related to carbohydrate metabolism and glycan biosynthesis in these systems, while qPCR showed higher strB1 abundance normalized to bacterial 16S rRNA gene abundance in soil and forage. These concurrent differences in soil properties, forage composition, and microbial communities were associated with bovine periodontitis prevalence. They do not demonstrate microbial transfer or causality but support the hypothesis that soil and forage conditions may influence environmental exposures related to oral microbiome dysbiosis.

**Graphical abstract:**

**Figure:**
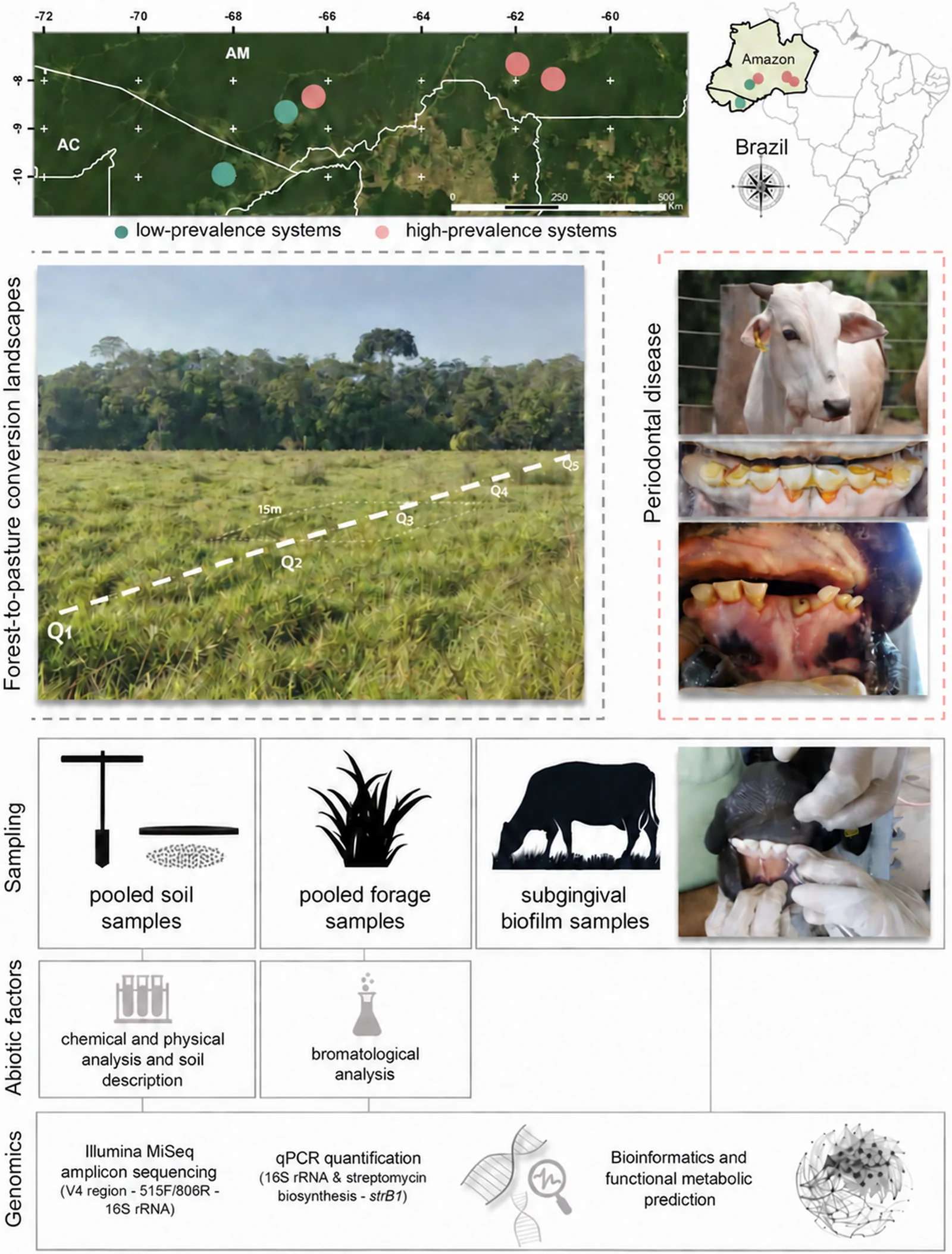

**Supplementary Information:**

The online version contains supplementary material available at https://doi.org/10.1007/s10653-026-03516-x.

## Introduction

Understanding the cascade of biotic and abiotic disturbances triggered by large-scale landscape simplification remains a major challenge, particularly when these changes manifest as health disorders in animals and humans (Gottdenker et al., 2014)., This issue is critical in tropical rainforests, where anthropogenic pressures on biodiversity continue to intensify (Newbold et al., 2014). Among the most consequential transformations is forest-to-pasture conversion, which drastically alters soil properties, nutrient balance, and microbial diversity (De Souza Braz et al., 2013; Nobre et al., 2016; Rocha et al., 2023). These changes may propagate across ecological networks and influence forage quality, with possible implications for cattle health, but this relationship remains poorly understood.

A relevant example is the neglected bovine oral disease historically known in Brazil as “swollen face”, which has been reported since the 1960s in different biomes, including the Amazon (Döbereiner et al., 2000). Periodontal diseases are multifactorial conditions associated with dysbiosis of the dental biofilm, progressing from reversible gingival inflammation to irreversible attachment loss when inflammation becomes persistent (Kinane et al., 2017; Papapanou et al., 2018). In cattle and other ruminants, periodontitis impairs dental function and has been associated with shifts in the oral microbiome (Borsanelli et al., 2018, 2022; Dutra & Borsanelli, 2022). Although the precise environmental triggers remain unclear, outbreaks of “swollen face” were historically reported after forest clearing and the establishment or renewal of pastures (Döbereiner et al., 2000). Earlier studies proposed that soil-associated antibiotic exposure, particularly to sub-inhibitory streptomycin concentrations, could facilitate the adherence of periodontal bacteria and biofilm formation (Grassmann et al., 1997; Kopp et al., 1996; Kumar & Ting, 2016). Other observations implicated changes in soil properties, forage mineral composition, and cattle nutrition. Long-term increases in soil pH and base saturation and changes in physical and hydraulic properties have been documented after forest-to-pasture conversion (De Souza Braz et al., 2013), while mineral supplementation and adequate Cu supply were associated with improved animal performance in affected herds (Moraes et al., 1994; Rosa & Döbereiner, 1994). These findings support several plausible environmental pathways but do not establish which factor initiates periodontal disease.

One Health recognizes that environmental, animal, and human health are interdependent and that changes in one component can affect the others (Banerjee & van der Heijden, 2023; Singh et al., 2023). In the pasture systems examined here, this framework can be represented as a hypothesized soil–plant-animal pathway: forest-to-pasture conversion modifies soil properties and microbial communities; these changes may affect forage composition and the microorganisms or metabolites ingested by grazing cattle; and repeated exposure may influence oral microbiome assembly and susceptibility to periodontitis. Soil and forage microbiomes containing taxa with known antagonistic potential, including Bacillus and Pseudomonas, could constrain the establishment of dysbiotic communities, but this suppressive role has not been demonstrated in the present system. Similarly, detection of strB1 indicates genetic potential for streptomycin biosynthesis rather than antibiotic production or exposure.

Building on this evidence, we investigated extensive livestock systems in the Western Brazilian Amazon to examine how soil properties, forage composition, and microbiome structure vary among pastures classified by herd-level prevalence of periodontal lesions. Our approach integrated environmental characterization with molecular profiling to evaluate soil physicochemical traits, forage nutritional attributes, and microbial communities across soil, forage, and bovine oral niches, alongside targeted quantification of the streptomycin biosynthesis gene. We hypothesized that high-prevalence pastures would exhibit greater disruption across the soil–plant-animal continuum, reflected in nutrient imbalances, differences in soil texture and pedogenic traits, and altered microbial diversity and interaction patterns, consistent with conditions that may favor oral dysbiosis. Conversely, low-prevalence pastures were expected to present more balanced edaphic conditions and microbial signatures hypothesized to be associated with environmental suppressiveness. We also evaluated whether disturbed systems showed increased potential for streptomycin biosynthesis in soil and forage as a potential, rather than demonstrated, contributor to conditions favoring pathogen adherence in the oral cavity.

By linking soil properties, forage composition, and multi-compartment microbial structure, this study provides an integrated ecological perspective on how post-conversion pasture conditions may influence cattle health and inform management strategies in already converted Amazonian landscapes.

## Material and methods

### Study area selection and sampling

The study was conducted in three regions of the Western Brazilian Amazon. Five extensive livestock farms were selected in Bujari (Acre), Boca do Acre, and Manicoré (Amazonas) (Table S1), based on recent deforestation reports and consultations with local institutions. Criteria included: (i) conserved Amazon Forest adjacent to pastures; (ii) no history of liming or chemical fertilization; and (iii) infrastructure for clinical examination and subgingival biofilm sampling. Soil and forage sampling followed Rocha et al. (2020) across eight pasture systems, with adjacent Amazon Forest bulk soil collected as a baseline reference. Linear transects (200 m) with five points spaced 50 m apart were established. At each point, bulk soil (0–10 cm) and forage leaves at grazing height were collected for abiotic and molecular analyses. Composite samples combined three subsamples per point. Material for molecular analysis was packed in sterile pouches, kept on ice, and stored at − 80 °C. Details on soil and bromatological analyses are provided in SI, Table S2 and Table S3.

### Criteria for cattle selection and examination

Adult beef cattle from herds of at least 200 animals, maintained for one year on extensive pasture (fed exclusively on pasture and mineral salt without additives, offered ad libitum), were included in the study. Herd selection for clinical examination was carried out in agreement with farm owners, following the predefined criteria. Based on clinical evaluations, farms were classified according to the prevalence of periodontal lesions in adult animals: high-prevalence class (HSL) when prevalence exceeded 20% of the herd, and low-prevalence class (LSL) when prevalence was below 5% (Table S1). Additional details on the clinical examination of cattle herds are provided in the SI.

### DNA extraction and amplicon sequencing

Soil DNA was extracted using the DNeasy PowerSoil kit (QIAGEN, Hilden, Germany) following the manufacturer’s protocol. Plant tissue DNA was obtained using a CTAB-based method (Doyle & Doyle, 1987), and bovine subgingival biofilm DNA using the GenElute Mammalian Genomic DNA Miniprep Kit (Sigma, USA). The V4 region of the 16S rRNA gene was amplified with barcoded primers 515F (5′-GTGCCAGCMGCCGCGGTAA-3′) and 806R (5′-GGACTACHVGGGTWTCTAAT-3′) (Apprill et al., 2015; Caporaso et al., 2012), and libraries were sequenced on an Illumina MiSeq platform (paired-end, 251 bp). Raw reads were demultiplexed in Python using primer barcodes. Quality filtering, denoising, ASV inference, and chimera removal were performed with DADA2 (Callahan et al., 2016, 2019); no separate adapter-removal program was used. Taxonomy was assigned using SILVA version 132 (Quast et al., 2012). Additional details are provided in the SI.

### qPCR quantification of 16S rRNA and streptomycin biosynthesis gene (*strB1*)

Quantitative real-time PCR (qPCR) was used to quantify 16S rRNA and strB1 genes in DNA from soil, forage, and bovine subgingival biofilms. The strB1 target was selected because it encodes a conserved component of the streptomycin biosynthetic pathway in Streptomyces spp. and provides a marker of genetic potential for streptomycin biosynthesis rather than antibiotic production. The 16S rRNA gene was amplified with primers 1055yF (5′-ATGGYTGTCGTCAGCT-3′) and 1392R (5′-ACGGGCGGTGTGTAC-3′) (Ritalahti et al., 2006); strB1 was amplified with strB1F (5′-ACTACGAGAGCCAGGAGCAGAT-3′) and strB1R (5′-TGACTCCGAGCTTGGTCAACT-3′), previously described by Tanaka et al. (2009). Reactions were performed in triplicate using SYBR® Green chemistry on a CFX96 system (Bio-Rad, USA), with standard curves generated from synthetic DNA standards. Thermal cycling conditions and detailed protocols are provided in the SI.

### Statistical analysis

#### Variable selection for soil and forage

To identify abiotic variables that best distinguished high-prevalence (HSL) from low-prevalence (LSL) systems, soil and forage chemical and physical properties were analyzed using an integrated feature-selection approach combining Least Absolute Shrinkage and Selection Operator (LASSO) and Random Forest. Random Forest was used as a supervised classification and variable-importance method, not as a differential-abundance test. Each method generated a subset of variables ranked according to its own importance criterion; these subsets were combined into a normalized feature-importance list, retaining variables with above-average scores. All abiotic variables were normalized using the min–max method before analysis (Patro & Sahu, 2015), and model stability was assessed over 1000 bootstrap iterations. Group differences for retained variables were evaluated separately using one-way analysis of variance as exploratory, unadjusted comparisons. Analyses were performed in R using glmnet and ranger (Friedman et al., 2010; Wright & Ziegler, 2017).

#### Metacommunity data analysis

Microbial community data were processed by quality filtering, denoising, and chimera removal, followed by exclusion of sequences classified as Chloroplast, Eukaryota, or Mitochondria. Samples with fewer than 1000 reads were excluded. The final dataset comprised 1,371,914 reads distributed across 11,470 amplicon sequence variants (ASVs), with an average of 13,450 reads per sample. Counts in each sample were scaled to the median library size as round[M × (x/Σx)], where x is the ASV count and M is the median sequencing depth; this procedure was not rarefaction. Core microbiota were defined as ASVs present in at least 50% of samples within each component (soil, forage, cattle) (Fig. S2). Community structure was visualized using nonmetric multidimensional scaling (NMDS) based on Bray–Curtis dissimilarities, and differences between herd-level prevalence classes were tested using PERMANOVA with approximately 10,000 permutations (Anderson et al., 2006). Indicator species analysis (IndVal) identified taxa with high specificity and fidelity for each prevalence class, with Benjamini–Hochberg adjustment of p-values. In the Random Forest workflow, taxa were screened using Kruskal–Wallis tests with false-discovery-rate adjustment and then ranked by classification importance using mean decrease in Gini. Random Forest importance was not interpreted as a differential-abundance statistic. Correlation heatmaps were used to explore associations between selected abiotic variables and ranked prokaryotic taxa. Visualization and additional analyses were supported by microeco (Liu et al., 2021), and analyses were conducted in R using phyloseq and vegan (McMurdie & Holmes, 2013; Oksanen et al., 2016). For a descriptive cross-compartment comparison, we matched exact 16S V4 ASV nucleotide sequences in the filtered pasture-soil, forage, and cattle datasets before ASV relabeling and median-depth scaling. Detection frequency and mean relative abundance, including zero counts, were calculated separately for each compartment.

#### Alpha and gamma diversities

Hill-number diversity profiles were evaluated separately within each component (soil, forage, and cattle) and herd-level prevalence class using the entropart package (Marcon & Hérault, 2015). Samples were assigned equal weights within each component-by-class metacommunity. Alpha diversity represented the mean effective diversity within samples, gamma diversity represented the effective diversity of the corresponding pooled metacommunity, and multiplicative beta diversity was defined as gamma/alpha. Profiles were calculated at q = 0 (ASV richness), q = 1 (exponential Shannon entropy), and q = 2 (inverse Simpson concentration) (Chao et al., 2014; Jost, 2006). Between-sample compositional differences were additionally evaluated using Bray–Curtis dissimilarities, NMDS, and PERMANOVA.

#### Co-occurrence network analysis

Correlation-based network analysis was used to describe microbial association patterns across and within soil, forage, and cattle components. Edges represented Spearman correlations between ASV abundance profiles. Associations were retained at *p* < 0.01, with correlation thresholds either optimized by the Random Matrix Theory procedure (Deng et al., 2012) or set conservatively for component-specific networks (|ρ| approximately 0.60–0.70); false-discovery-rate adjustment was applied where specified in the component analysis. Separate networks were constructed for high-prevalence (HSL) and low-prevalence (LSL) systems, and genus-level networks were used to examine core microbiota. Network metrics were summarized at the network level or, for node-level measures, across nodes within each network. Correlations indicate statistical co-occurrence and were not interpreted as direct ecological interactions. Analyses used microeco, and networks were visualized with the Fruchterman-Reingold layout in Gephi (Bastian et al., 2009).

#### Prediction of metabolic profiles

Functional potential was predicted from 16S rRNA profiles to compare soil, forage, and cattle microbiomes between high-prevalence (HSL) and low-prevalence (LSL) systems. Tax4Fun (Aßhauer et al., 2015) mapped SILVA SSU Ref 123 NR taxonomic profiles to KEGG Orthologs and pathway annotations. Analyses first selected KEGG hierarchy level 2 categories, including “Carbohydrate Metabolism,” “Biosynthesis of Other Secondary Metabolites,” and “Glycan Biosynthesis and Metabolism,” and then evaluated the corresponding level 3 pathways. Differences in predicted pathway representation were assessed using LEfSe with α = 0.01, false-discovery-rate adjustment, 999 bootstrap iterations, and an LDA score threshold of 3 (Segata et al., 2011). These results represent predicted functional potential rather than measured gene expression or metabolite production.

#### Quantitative real-time PCR

Threshold cycle (ct) values were averaged across qpcr triplicates. For soil and forage, strb1 absolute abundance was divided by bacterial 16S rRNA gene abundance and is therefore reported as 16 s-normalized strb1 abundance, not as the proportion of bacteria carrying strb1. For the forest-to-pasture soil dataset, the log-transformed strb1/16S rRNA ratio was modeled as a function of soil sum of bases (SB), herd-level prevalence class, and their interaction, with sample included as a random intercept. Type II Wald f-tests were used after residual diagnostics. Separate mixed-effects models evaluated prevalence-class differences in pasture soil and forage. In bovine subgingival biofilms, absolute abundances of strb1 and 16S rRNA genes were compared between clinically diseased and clinically healthy animals. Statistical analyses were performed in R using lme4 and car.

## Results

### Soil and forage properties associated with microbiota composition

**Fig. 1:**
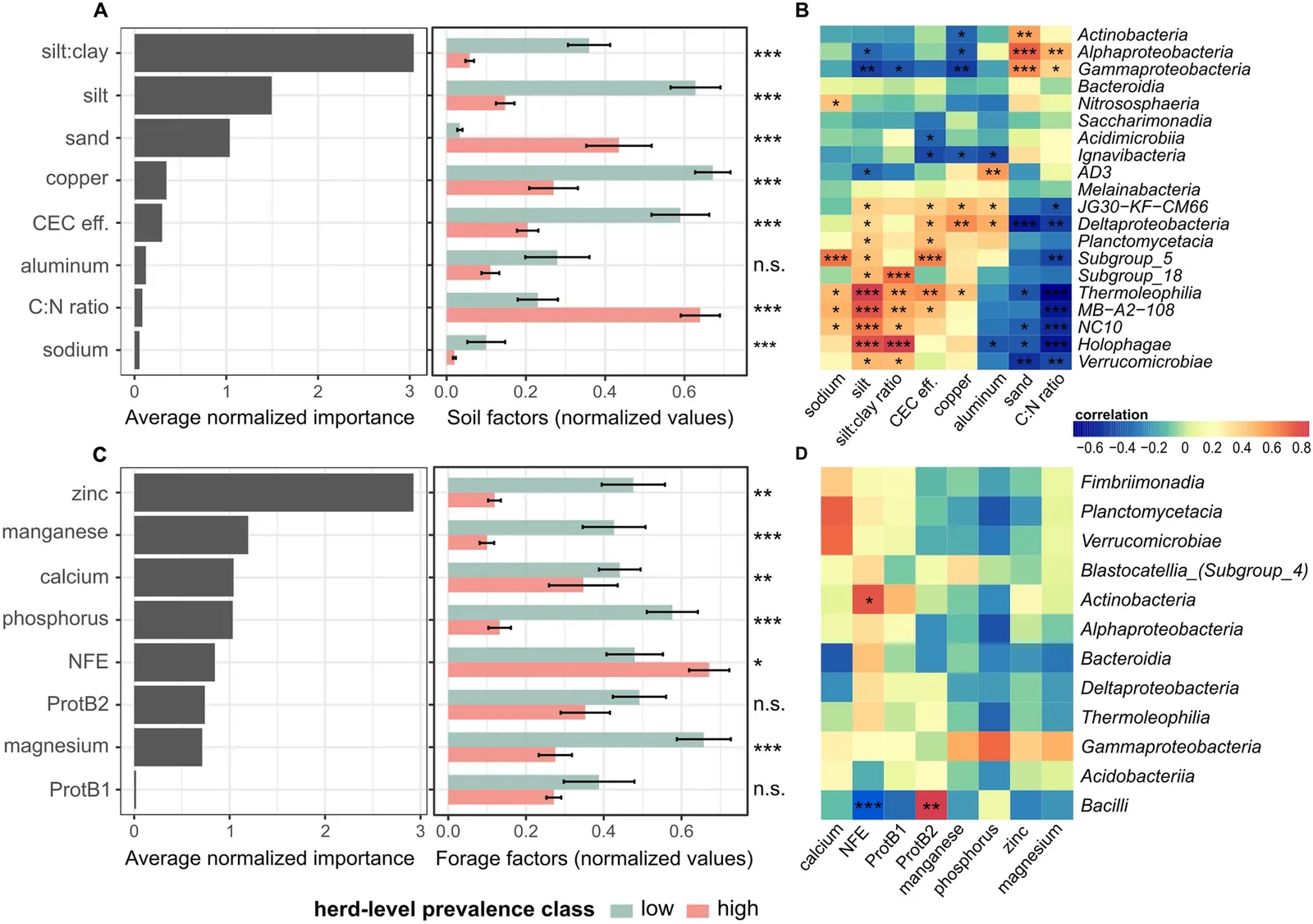
Abiotic variables of **A)** soil and **C)** forage selected by the integrated Random Forest-LASSO feature-selection model. Values were min-max normalized before separate one-way ANOVA comparisons; the displayed *p*-values are nominal and unadjusted. Bars represent the magnitude of each variable in HSL and LSL systems. Significance levels: (***) *p* < 0.001, (**) *p* < 0.01, (*) *p* < 0.05, n.s. not significant. Error bars indicate ± standard error (SE) (n = 5 systems per class). Heatmaps show Spearman correlations between selected abiotic variables and bacterial taxa in **B)** soil and **D)** forage. *Abbreviations*: CEC eff., effective cation exchange capacity; NFE, nitrogen-free extract; ProtB2, insoluble protein (intermediate); ProtB1, soluble protein

Soil properties differed between herd-level prevalence classes, with LSL pastures showing finer texture and higher fertility-related variables than HSL pastures. After normalizing importance scores from the integrated feature-selection approach combining Random Forest and LASSO, key soil and forage variables were retained (Fig. 1A and C; Fig. S1). The reported p-values were obtained from separate one-way ANOVA comparisons of the retained variables and are nominal, unadjusted values used for exploratory interpretation. In LSL pastures, silt:clay ratio (*p* < 0.001; ~4.4-fold), silt content (*p* < 0.001; ~3.2-fold), Cu (*p* < 0.001; ~2.1-fold), effective CEC (*p* < 0.001; ~1.6-fold), and Na (*p* < 0.001; ~3.4-fold) were higher. Forage traits with greater importance included Zn (*p* = 0.002; ~1.6-fold), Mn (*p* < 0.001; ~2.8-fold), Ca (*p* = 0.006), P (*p* < 0.001), Mg (*p* < 0.001), and protein fractions (ProtB2 and ProtB1). Conversely, HSL soils had greater sand content (*p* < 0.001; ~7.4-fold) and C:N ratio (*p* < 0.001), while forage showed increased nitrogen-free extract (NFE; *p* = 0.040). Microbial correlations mirrored these patterns (Fig. 1B and D). Actinobacteria (Thermoleophilia), Acidobacteria (Holophagae), and Rokubacteria were positively correlated (Spearman's ρ > 0.60; *p* < 0.001) with silt content and silt:clay ratio, but negatively correlated with C:N ratio. Alphaproteobacteria correlated with sandy soils, while Acidobacteria (Subgroup 5) aligned with Na and CEC. In forage, Bacilli (Firmicutes) correlated positively with insoluble protein (ProtB2) and negatively with NFE, whereas Actinobacteria showed a positive correlation with NFE.

### Structure and diversity of microbiota along the soil-plant-animal continuum

**Fig. 2:**
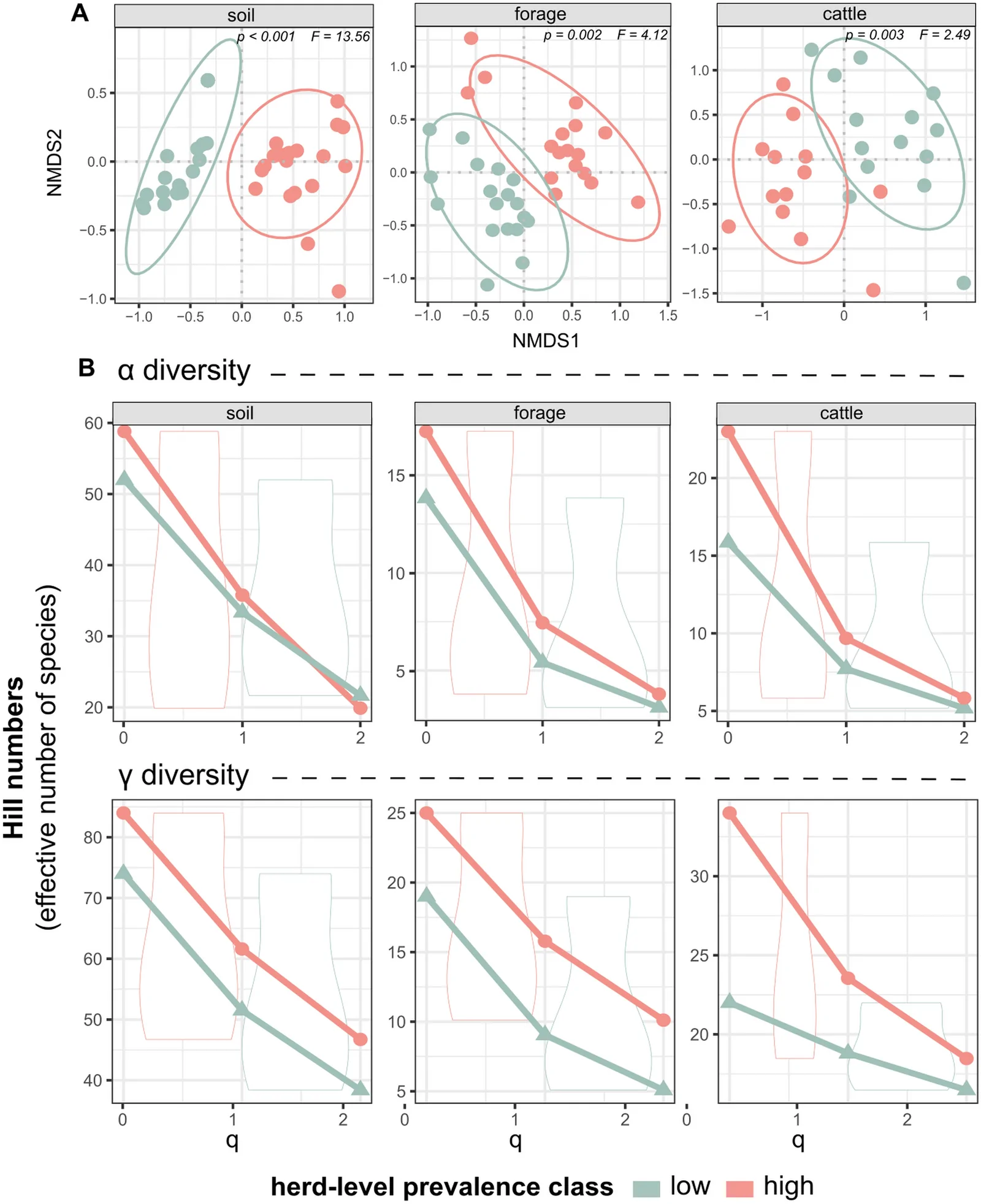
**Non-metric multidimensional scaling (NMDS) based on Bray–-Curtis dissimilarities of scaled core-microbiota ASV data from soil (HSL/LSL, 20/20), forage (17/18), and cattle (13/14).** Differences between herd-level prevalence classes were assessed using PERMANOVA with approximately 10,000 permutations. Hill-number profiles show mean alpha and gamma diversity; samples received equal weights within each component-by-class metacommunity, and multiplicative beta diversity corresponds to gamma/alpha. Orders were q = 0 (ASV richness), q = 1 (exponential Shannon entropy), and q = 2 (inverse Simpson concentration)

Microbial community structure differed between herd-level prevalence classes across the three compartments. NMDS ordination based on Bray–Curtis distances separated HSL and LSL communities in soil (HSL, n = 20; LSL, n = 20), forage (HSL, n = 17; LSL, n = 18), and cattle (HSL, n = 13; LSL, n = 14) (Fig. 2A). PERMANOVA supported differences between prevalence classes for soil (R2 = 0.263, *p* < 0.001), forage (R2 = 0.111, *p* = 0.002), and cattle (R2 = 0.091, *p* = 0.003). Hill-number profiles provided complementary information. Mean alpha diversity was generally higher in HSL systems across q = 0, 1, and 2, except at q = 2 in soil, which was slightly higher in LSL. Gamma diversity was consistently greater in HSL systems for all components (Fig. 2B); multiplicative beta diversity is represented by the corresponding gamma/alpha relationship.

### Bacterial community signature of HSL and LSL systems

#### Differential abundance and indicator species of the core microbiota

**Fig. 3:**
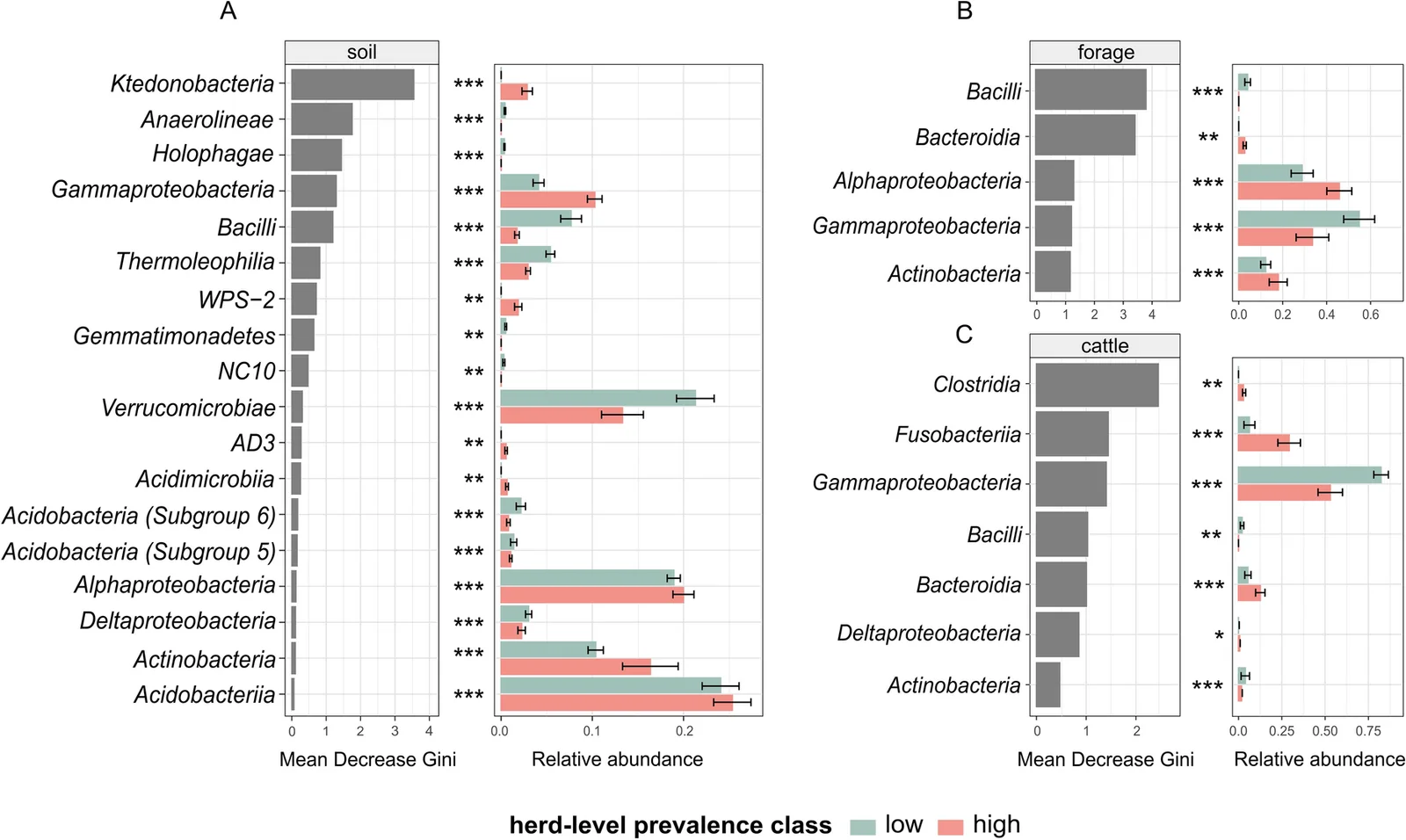
Relative abundance and Random Forest feature importance of bacterial classes associated with herd-level prevalence class in **A)** soil, **B)** forage, and **C)** cattle. Classes are ordered by Random Forest mean decrease in Gini importance, which is a ranking metric rather than a differential-abundance statistic. Significance symbols in the relative-abundance panels derive from Kruskal-Wallis tests with false-discovery-rate adjustment: (***) FDR-adjusted *p* < 0.001, (**) *p* < 0.01, (*) *p* < 0.05, n.s. not significant

Kruskal-Wallis comparisons with false-discovery-rate adjustment, indicator-species analysis, and Random Forest feature ranking provided complementary, but distinct, evidence for taxa differentiating herd-level prevalence classes across soil, forage, and cattle compartments (Fig. 3; Fig. S3; Table S4). Random Forest Gini importance was used only for ranking and was not interpreted as a differential-abundance statistic. In soil, HSL pastures were depleted in Bacillus, Gaiellales, and Candidatus Solibacter, while enriched in Burkholderia-Caballeronia-Paraburkholderia, Leifsonia, and Acidobacteria-related genera. HSL forage was similarly depleted in Bacillus and Pseudomonas, but enriched in Spirosoma, Xanthomonas, and Sphingomonas. In cattle, HSL microbiota was enriched in Bacteroidetes and Fusobacteria, whereas LSL systems were enriched in Gammaproteobacteria and Firmicutes, including Streptococcus and Corynebacterium. Overall, Bacilli were more abundant in LSL systems, while Bacteroidia predominated in HSL components. Indicator species analysis further supported these patterns, identifying taxa with high specificity for each prevalence level, including Leifsonia in HSL soil and ADurb.Bin063-1 in LSL soil, Spirosoma in HSL forage and Curtobacterium in LSL forage, and Caviibacter in HSL cattle and Streptococcus in LSL cattle (Table S4). Exact V4-sequence comparison identified 17 ASVs shared among soil, forage, and cattle samples (Table S5). Their combined mean relative abundance was 0.570% in soil, 8.884% in forage, and 2.042% in cattle. Within prevalence classes, nine ASVs were shared across all three HSL compartments and six across all three LSL compartments; these descriptive counts were not formally compared. Only five of the pooled ASVs occurred in at least two samples per compartment, and none occurred in at least half of samples in all three. This limited overlap describes sequence co-detection, not microbial transmission.

#### Co-occurrence network of the prokaryotic metacommunity

**Fig. 4:**
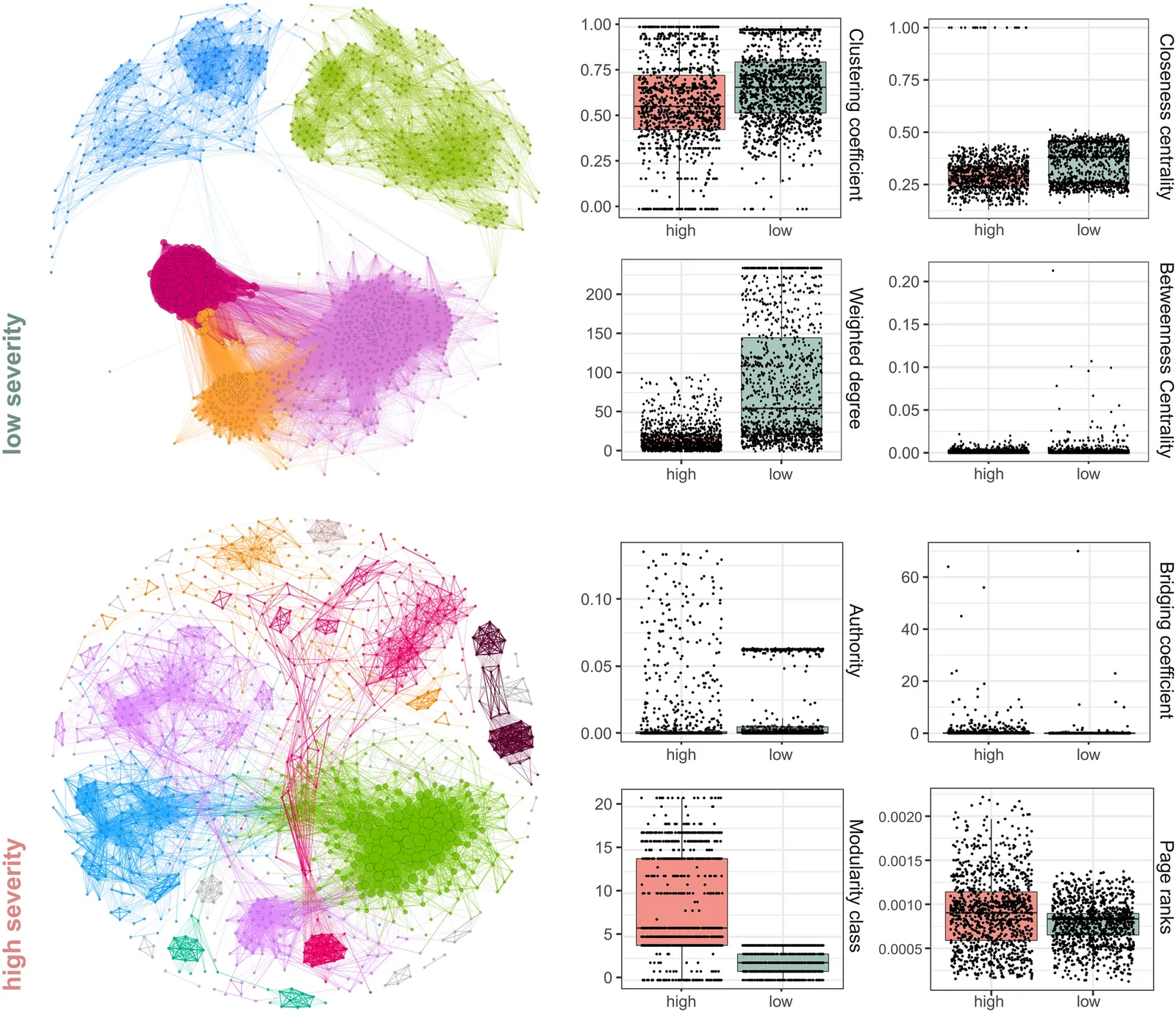
**Correlation networks constructed from the combined soil, forage, and cattle ASV dataset for LSL and HSL systems.** Nodes represent ASVs, edges represent retained Spearman correlations, and colors indicate categorical module membership. Only associations with *p* < 0.01 were retained; correlation thresholds were optimized by Random Matrix Theory (LSL = 0.53; HSL = 0.69). The pooled construction permits cross-compartment co-occurrence patterns but does not demonstrate microbial transfer or direct ecological interaction

Correlation-network topology differed between prevalence classes (Fig. 4; Table S6). Network-level modularity was higher in HSL than LSL across the three components, whereas the number of modules differed only in soil. The numbers of nodes and edges and average clustering varied by component, without a uniform HSL-versus-LSL pattern. Node-level centrality measures were summarized across nodes within each network, and module-class labels were treated only as categorical membership. These descriptive differences indicate contrasting association structures and were not interpreted as direct evidence of ecological stability or suppressiveness. Complete network metrics are provided in Table S6.

#### Keystone taxa of the core microbiota

Network analysis identified highly central taxa within the core microbiota across soil, forage, and cattle compartments (Fig. S5; Table S6). In soil, approximately half of the LSL edges were positive, and high-betweenness nodes included Bradyrhizobium and Bacillaceae; HSL soil networks had a higher proportion of positive correlations and included central nodes such as Conexibacter and HSB_OF53-F07. In forage, prominent correlations involved Pseudomonas and Methylobacterium in LSL and Sphingomonas and Spirosoma in HSL. In cattle, central taxa included Neisseria and Corynebacterium in LSL, whereas prominent HSL correlations included Porphyromonas and Fusobacterium. These edges represent statistical co-occurrence patterns rather than demonstrated ecological interactions.

### Differences in predicted metabolic pathways between HSL and LSL systems

Tax4Fun-predicted functional profiles differed between prevalence classes, showing parallel differences across soil, forage, and cattle microbiomes rather than demonstrating functional transfer among compartments (Fig. S6). Using LEfSe criteria of FDR-adjusted p < 0.01 and LDA score ≥ 3, LSL microbiomes showed higher predicted representation of pathways related to secondary-metabolite biosynthesis, including beta-lactam and alkaloid biosynthesis. HSL forage and cattle microbiomes showed higher predicted representation of pathways associated with phenylpropanoid and streptomycin biosynthesis, while starch and sucrose metabolism was more highly represented across the three HSL compartments. HSL cattle also showed higher predicted representation of amino-sugar and nucleotide-sugar metabolism. Among glycan-related predictions, HSL soil showed lower representation of glycosaminoglycan and N-glycan biosynthesis and higher representation of peptidoglycan biosynthesis, whereas HSL cattle showed higher predicted representation of lipopolysaccharide biosynthesis (LDA = 4.0) and glycan degradation (LDA = 3.77). These predictions do not establish gene expression, metabolite production, or cross-compartment transmission.

### Abundance of the streptomycin biosynthesis gene (strB1)

**Fig. 5:**
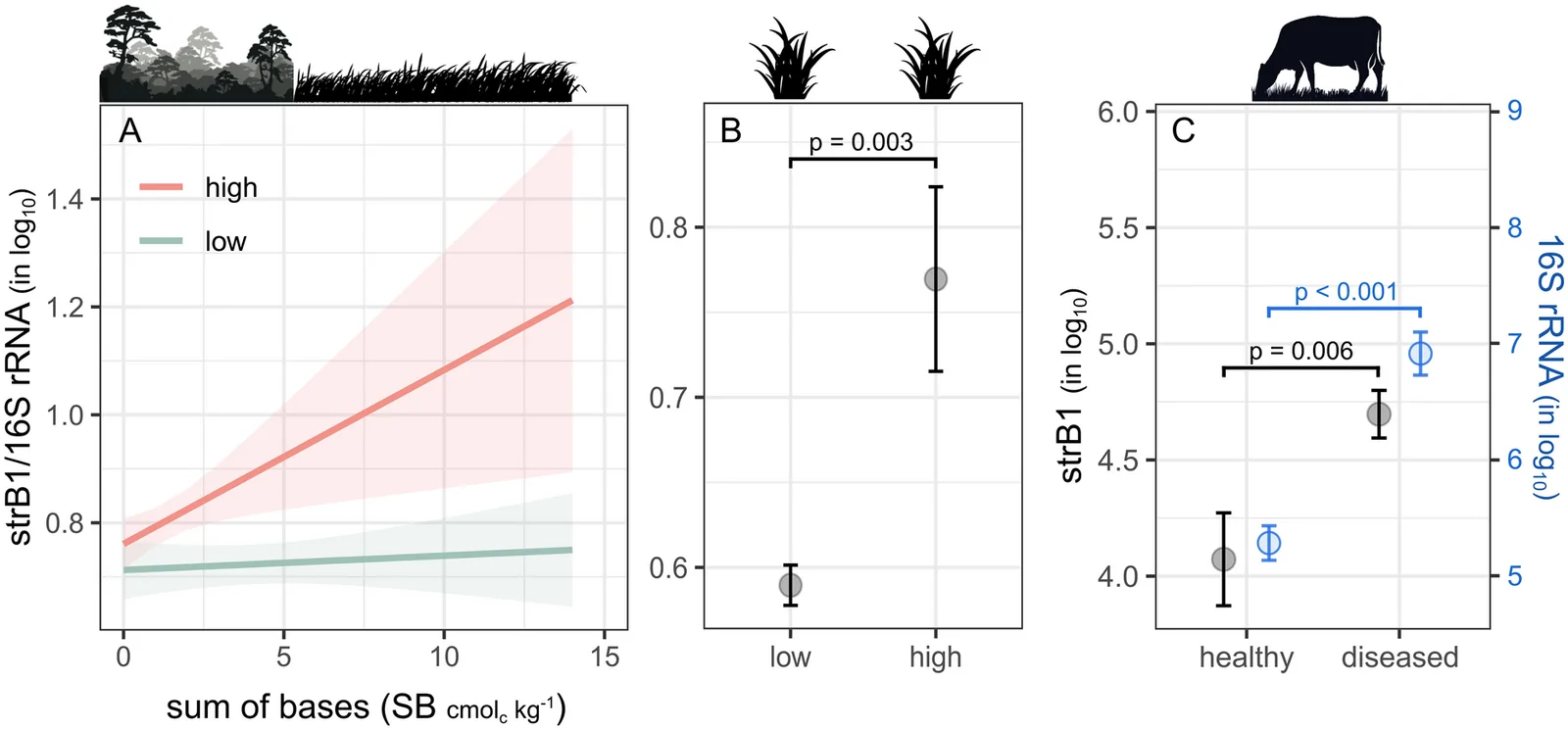
**qPCR quantification of strB1 across study compartments.** **A)** Relationship between soil sum of bases (SB) and 16S-normalized strB1 abundance in forest and pasture soils, shown by herd-level prevalence class; **B)** 16S-normalized strB1 abundance in forage; and **C)** absolute strB1 and bacterial 16S rRNA gene copy numbers in subgingival biofilms from clinically diseased and clinically healthy cattle.

qPCR showed higher 16S-normalized strB1 abundance in HSL soil and forage samples (Fig. 5). In the forest-to-pasture soil dataset, prevalence class was associated with the strB1/16S rRNA ratio (F = 12.56, *p* < 0.001). The main effect of soil sum of bases (SB) was not significant (F = 2.10, *p* = 0.153), but the SB by prevalence-class interaction was significant (F = 4.86, *p* = 0.031), reflecting a positive association in HSL soils that was not observed in LSL soils (Fig. 5A). The ratio was also higher when pasture soil was evaluated separately (F = 6.85, *p* = 0.013) and in forage (F = 10.14, *p* = 0.003; Fig. 5B), indicating greater genetic potential for streptomycin biosynthesis without demonstrating antibiotic production. The taxonomic source of this signal was not directly established. In bovine subgingival biofilms, clinically diseased animals had higher absolute copy numbers of both strB1 and bacterial 16S rRNA genes than clinically healthy animals (strB1: F = 9.07, *p* = 0.006; 16S rRNA: F = 37.08, *p* < 0.001; Fig. 5C). Because a cattle strB1/16S ratio was not evaluated, this result indicates greater absolute gene abundance rather than relative strB1 enrichment.

## Discussion

### Soil and forage conditions associated with herd-level periodontitis prevalence

Our analysis revealed a pattern of associations in which abiotic disturbance and nutritional imbalance were linked to microbial reorganization across the soil–plant-animal continuum, with potential implications for oral dysbiosis and infection risk in cattle. Low-prevalence (LSL) systems consistently showed higher soil fertility and more balanced forage nutritional profiles, whereas high-prevalence (HSL) systems exhibited elevated soil C:N ratios, sandier texture, and reduced macro and micronutrient availability. These patterns suggest that herd-level periodontitis prevalence was associated with a broader edaphic and nutritional context, rather than with a single environmental variable.

As the duration of feeding and diet composition can modulate periodontal disease severity (Dutra et al., 1993; Döbereiner et al., 2000; Saraiva et al., 2023), the micronutrient signal in our integrated model is particularly relevant because Zn was nearly twice as abundant in LSL forage, and Cu was among the most important soil variables. Deficiencies in these elements have been linked to anemia, osteodystrophy, and increased infection risk (Tessman et al., 2001), while Zn deficiency has been associated with oral disease risk (Orbak et al., 2007; Uwitonze et al., 2020). Historical trials reported that Cu supplementation reduced mortality and improved weight gain in affected calves (Souza et al., 1985). Ecologically, Zn supports plant defense, while Cu compounds are widely used for crop protection and are involved in plant stress responses (Cabot et al., 2019; Hasanuzzaman et al., 2017; Lamichhane et al., 2018). Together with higher Ca, Mg, K, Fe, and Mn in LSL forage, these findings suggest an association between balanced soil–plant nutrition and lower disease severity, rather than a single-element causal explanation. Previous work on bovine supragingival biofilm also suggests that mineral profiles may differ between healthy and diseased oral conditions, reinforcing the need to consider nutrition and metabolic disorders within the soil–plant-animal continuum (Saraiva et al., 2020).

### Microbial community reassembly and functional shifts

High-prevalence (HSL) systems showed greater alpha and gamma diversity across soil, forage, and cattle microbiota, a pattern that can accompany dysbiotic expansion in periodontal systems but does not, by itself, indicate reduced ecological stability (Borsanelli et al., 2018, 2022; Scannapieco & Dongari-Bagtzoglou, 2021). Non-metric multidimensional scaling (NMDS) showed differences in community composition between herd-level prevalence classes, while network topology varied among compartments. HSL networks had higher network-level modularity, but node number, edge number, and clustering did not follow a uniform HSL-versus-LSL pattern. Together, the diversity and network results indicate microbial community reorganization associated with high-prevalence systems. Because correlation-network metrics are descriptive and system-specific, they were not used as direct measures of ecological stability, resilience, or suppressiveness (Banerjee et al., 2019).

The observed community differences may reflect environmental filtering associated with soil texture, nutrient balance, and forage composition, although the study was not designed to partition deterministic and stochastic assembly processes. This interpretation is consistent with ecological literature describing how both processes and priority effects can shape microbial communities (Debray et al., 2021; Dini-Andreote et al., 2015; Mo et al., 2024; Nemergut et al., 2013; Tripathi et al., 2018). The prevalence-associated core structures and centrality patterns are therefore treated as signatures of community reorganization rather than evidence of a specific assembly mechanism or resilience response. Forage resource profiles also differed, with slightly greater nitrogen-free extract (NFE) in HSL systems and higher predicted representation of starch and sucrose metabolism in HSL soil, forage, and cattle microbiomes. Experimental studies in other systems show that carbohydrate availability can influence host-associated microbial communities (Morimoto et al., 2019; Petri et al., 2020). In the present observational dataset, the parallel NFE and predicted functional differences support an association with microbial reorganization but do not establish a shared causal process across compartments.

Importantly, carbohydrate metabolism alone should not be interpreted as a universal marker of disease, since similar functions may also reflect dietary adaptation and normal host-associated microbial activity. Its relevance here lies in its co-occurrence with glycan biosynthesis and metabolism, including lipopolysaccharide biosynthesis and glycan degradation, as reported in chronic human periodontitis (Wang et al., 2013). In cattle with periodontitis, protein families linked to chemotaxis, flagellar assembly, bacterial motility, proteases, and hydrolases further suggest functional potential related to motility, adhesion, and tissue degradation (Borsanelli et al., 2022). Together, these patterns suggest that carbohydrate enrichment may become relevant to oral dysbiosis when embedded in a broader functional context associated with biofilm restructuring, inflammation, and host tissue interaction.

Differential-abundance and indicator analyses showed lower representation of Bacillus and Pseudomonas in HSL cores and greater representation of these genera in LSL forage and cattle. Some members of these genera have recognized antagonistic activity and metabolite-production capacity (Raaijmakers & Mazzola, 2016; Sun et al., 2021) and have been associated with healthy ruminant oral microbiota (Borsanelli et al., 2018; Borsanelli et al., 2021, Borsanelli et al., 2023). However, these functions were not measured here, and the observed network correlations cannot establish antagonism or disease suppressiveness. The results therefore identify prevalence-associated taxa and co-occurrence patterns that warrant direct functional testing.

HSL components also showed greater representation of taxa previously associated with environmental stress, anaerobic niches, or dysbiotic biofilms. Actinobacteria, Clostridiales, and Bacteroidetes were prominent in HSL components, while HSL forage networks contained frequent positive correlations involving Sphingomonas and Spirosoma and HSL cattle networks included correlations between Porphyromonas and Fusobacterium. Porphyromonas and Fusobacterium are recognized members of dysbiotic oral communities in ruminants with periodontitis (Borsanelli et al., 2018; Borsanelli et al., 2023; Silva et al., 2019). Nevertheless, positive network correlations indicate co-occurrence only and do not demonstrate cooperation, biofilm formation, or cross-compartment transfer.

### Streptomycin biosynthesis potential and biofilm-associated dysbiosis

We therefore examined whether high-prevalence systems showed greater genetic potential for streptomycin biosynthesis across the sampled compartments. qPCR showed higher 16S-normalized strB1 abundance in HSL soil and forage. In the forest-to-pasture soil dataset, the association between the strB1/16S rRNA ratio and soil sum of bases differed by prevalence class, with a positive relationship in HSL soils but not in LSL soils. These patterns are compatible with, but do not directly identify, environmental microbial groups carrying streptomycin-biosynthesis genes. Previous studies reported increased Actinobacteria after forest-to-pasture conversion in the Amazon (Petersen et al., 2019; Rocha et al., 2020) and showed that streptomycin can affect adherence of periodontal bacteria under experimental conditions (Döbereiner et al., 2000; Grassmann et al., 1997; Kopp et al., 1996). In the present study, however, neither streptomycin production nor animal exposure was measured. The parallel strB1 and predicted functional signals should therefore be interpreted as hypothesis-generating evidence of biosynthetic potential.

Ingestion of forage and associated soil particles is a plausible route by which cattle encounter environmental microorganisms and metabolites (Attwood et al., 2019; Banerjee & van der Heijden, 2023). We observed higher absolute abundances of both strB1 and 16S rRNA genes in subgingival biofilms of diseased cattle, but this does not demonstrate relative strB1 enrichment, environmental origin, or exposure to streptomycin. Source tracking and shared-strain analyses were not performed, and the sampling design cannot resolve microbial transmission among soil, forage, and cattle. Thus, the environmental exposure pathway remains a hypothesis to be tested using paired source-sink sampling, strain-resolved metagenomics, and direct chemical measurements.

### Management implications for environmental and animal health

From an environmental management perspective, these findings align with recent calls to explicitly include soil microbiomes in One Health policy and decision-making (Singh et al., 2023). First, micronutrient stewardship, particularly maintaining balanced Zn and Cu availability in soils and forage, may help support plant defense, animal nutrition, and resistance to infection (Cabot et al., 2019; Hasanuzzaman et al., 2017; Lamichhane et al., 2018; Orbak et al., 2007; Souza et al., 1985; Uwitonze et al., 2020). Second, practices that limit erosion and maintain vegetation cover may reduce the transfer of soil particles and soil-associated microorganisms to forage (Attwood et al., 2019). These include rotational grazing, pasture renovation, buffer strips, and diversified sward management. Third, microbiome-aware pasture management practices, including reduced disturbance, organic amendments, diverse swards, and balanced nutrient inputs, may help preserve microbial groups associated with suppressive functions and reduce selection for antibiotic-producing guilds (Berg et al., 2015; Raaijmakers & Mazzola, 2016). Such practices may also help avoid forage conditions that favor readily fermentable substrates and dysbiotic transitions (Petri et al., 2020). Fourth, surveillance strategies that incorporate qPCR markers, such as strB1*,* along with targeted functional profiling and microbial network indicators, could contribute to early detection of high-risk environmental conditions and dysbiotic transitions. In parallel, routine oral clinical examination of animals should be considered as part of integrated herd health monitoring, enabling early detection of periodontal lesions and facilitating timely intervention.

### Limitations and future directions

Because microbial functional diversity can influence important soil and host-associated processes, the predicted functional differences observed here should be interpreted as indicators of potential rather than direct measurements of activity. Tax4Fun predictions depend on available reference genomes and may be less accurate for poorly characterized environmental and host-associated taxa. These predictions should therefore be validated using shotgun metagenomics, metabolomics, proteomics, targeted culture, or direct chemical assays. The associations observed across compartments are also correlative, and source tracking was not performed; consequently, shared patterns cannot be interpreted as microbial transmission.

The study included five farms and eight pasture systems selected to provide a clear contrast between HSL and LSL conditions. This design supported the integrated comparison but limits inference about subtler effects, continuous prevalence gradients, and the broader Amazonian pasture landscape. Host and management variables, including breed, age, sex, body condition, dental wear, and recent treatments, were not consistently available and could not be fully controlled. Future studies should include more systems, model prevalence continuously, incorporate paired source-sink sampling, and use longitudinal or controlled designs to evaluate micronutrient balance, forage quality, soil-particle exposure, microbial assembly, land-use history, and seasonality.

## Conclusion

Our findings support an integrated framework in which soil and forage characteristics,including micronutrient availability, NFE, and soil texture,were associated with microbial diversity, composition, and network topology across soil, forage, and cattle compartments. High-prevalence systems also showed distinct prevalence-associated taxa, predicted differences in carbohydrate and glycan metabolism, and higher 16S-normalized strB1 abundance in soil and forage. These parallel patterns identify environmental and microbial features associated with bovine periodontitis prevalence, but do not demonstrate microbial transfer, streptomycin production, or causality. In converted Amazonian pasture landscapes, the results support further evaluation of integrated soil, forage, and herd-health monitoring and provide testable hypotheses for longitudinal and experimental studies of environmental contributions to bovine oral dysbiosis.

## Supplementary Information

Below is the link to the electronic supplementary material.

Supplementary file1 (PDF 1678 kb)

## Data Availability

The datasets generated and analyzed in this study are available in online repositories. Soil data can be accessed at MG-RAST (mgp94905). Forage and cattle data are available in the NCBI Sequence Read Archive under BioProject accession number PRJNA1469195 and PRJNA701760, respectively.
