# Supplementary material for: Soil-plant-animal properties and microbial reassembly distinguish cattle oral disease prevalence across converted Amazon pasture systems
Source: Environ Geochem Health. 2026 Oct 7;48(16):627. doi: 10.1007/s10653-026-03516-x (PMC13645925; doi:10.1007/s10653-026-03516-x)
Supplement: Supplementary file 1 [file 10653_2026_3516_MOESM1_ESM.pdf]

Supplemental information for:

Soil-plant-animal properties and microbial reassembly distinguish cattle oral disease prevalence across converted Amazon pasture systems

Fernando Igne Rocha[a,b*], Ana Carolina Borsanelli[c], Aline Pacobahyba de Oliveira[d], Cecílio Viega Soares Filho[e], Marcia Reed Rodrigues Coelho[f], Stefan Schwab[f], Carlos Magno dos Santos[f,g], Wenceslau Geraldes Teixeira[d], James Cole[h], Adina Howe[i], Iveraldo Santos Dutra[e], Ederson da Conceição Jesus[f]

[a] Department of Soil, Federal Rural University of Rio de Janeiro, Seropédica, RJ, Brazil

[b] Center for Nuclear Energy in Agriculture, University of São Paulo, Piracicaba, SP, Brazil

[c] Department of Veterinary Medicine, Federal University of Goiás, Goiânia, GO, Brazil

[d] National Soil Research Center, Embrapa Soils, Rio de Janeiro, RJ, Brazil

[e] Department of Production and Animal Health, São Paulo State University, Araçatuba, SP, Brazil

[f] National Agrobiology Research Center, Embrapa Agrobiologia, Seropédica, RJ, Brazil

[g] SENAI Innovation Institute for Green Chemistry, Rio de Janeiro, RJ, Brazil

[h] Department of Plant, Soil and Microbial Sciences, Michigan State University, East Lansing, MI, USA

[i] Department of Agricultural and Biosystems Engineering, Iowa State University, Ames, IA, USA

Supplemental information includes:

- Supplementary material and methods
- Table S1 to S6
- Figure S1 to S6

Supplementary material and methods

1. Soil classification and characterization

The soils from all studied areas were classified, using one profile per land-use where morphological characterization and horizon soil sampling were carried out according to Santos et al. (2005). Soil physical attributes (particle size distribution and flocculation degree) were determined by the sedimentation method and reading by densimeter from the sample dispersion with 0.1 mol L–1 sodium hydroxide solution. The chemical analyses consisted of pH in water and KCl 1 mol L–1, determined potentiometrically, in the soil: 1:2.5 solution with 1h of contact and agitation of the suspension at the time of reading. Exchangeable sodium and potassium (Na+ and K+) were extracted with HCl 0.5 mol L^{-1} + H2SO4 0.0125 mol L^{-1} (Mehlich^{-1}), in the proportion of 1:10 and determined by photometry of flame emission.

The measurement of exchangeable calcium and magnesium (Ca^{2+} and Mg^{2+}) was performed by atomic absorption spectroscopy and exchangeable aluminum (Al^{3+}) by titration after extraction with KCl 1 mol L–1 in the proportion of 1:10. The determination of potential acidity (H + Al) was carried out by titration after extraction with calcium acetate 0.5 mol L–1 in the proportion 1:10 and pH 7.0. The organic carbon was determined by titration of the remaining potassium dichromate with ammoniacal ferrous sulfate after the oxidation process. The calculation of derived correlations, i.e., sum of bases (SB = Ca^{2+} + Mg^{2+} + K^{+}), base saturation index (BS% = 100 × SB/total cation exchange capacity (CEC)), and Al saturation index (m% = (mmolc (Al^{3+}) dm^{-3} × 100)/(mmolc (effective CEC) dm^{-3})), were also analyzed (Teixeira et al., 2017) at the National Soil Research Center/ Embrapa Solos, Brazil.

The soil profiles were classified following the criterion of the Brazilian System of Soil Classification (Santos et al., 2005) and World Soil Reference Base (WRB, 2015a).

2. Bromatological analysis

Total nitrogen (N) content was determined by sulfuric digestion followed by distillation using the micro-Kjeldahl method, as described by (AOAC, 1995). Crude protein content was calculated by multiplying total N by 6.25 (Silva and Queiroz, 2002). Concentrations of macronutrients (N, P, K, Ca, Mg, and S) and micronutrients (Zn, Fe, Mn, and Cu) were determined according to (Malavolta, 1997). Fiber fractions, including insoluble neutral detergent fiber (INDF) and insoluble acid detergent fiber (IADF), were analyzed following (Van Soest; Robertson; Lewis, 1991), with adjustments for a Tecnal TE-149

fiber tester. After INDF determination, residual fiber was incubated for ADF analysis. In vitro dry matter digestibility (DMD) was assessed using the protocol described by (Tilley; Terry, 1963), modified by (Van Soest; Wine; Moore, 1966), in a Daisy II-ANKOM® incubator (Ankom Technology Corporation Fairport, NY, USA), with ruminal fluid from cattle fed tropical grasses. Additional parameters were determined according to (Silva; Queiroz, 2002). Information on bromatological characterization can be found on **Table S4.**

3. Cattle herds and intra-oral clinical examination

The criteria for inclusion of herds in the study were having adult cattle kept at least 1 year in the pastures and animal diet should be based only of extensive pasture, natural water, and mineral supplement. Cattle with clinical signs of other infectious or parasitic diseases or that received antibiotics up to 3 months before the start of the study were excluded. The sampling criteria to assess the occurrence of periodontal lesions in the 12 selected herds were established by adopting the prevalence of 20% of periodontal lesions in herds (Borsanelli et al., 2016) and a 95% confidence interval. Thus, it would be necessary to evaluate at least 162 animals in the six regions studied, according to the formula for determining the sample size based on the estimate of the proportion to the population (Miot, 2011).

The animals were selected as a convenience sample as it passed down the examination line and the intra-oral and periodontal evaluation by sampling was carried out by mechanical restraint of the animals, with the aid of a mouth opener, flashlight, and Williams periodontal probe. For the purposes of this study, the periodontal clinical examination of the incisor teeth (404-401, 301-304; labial and lingual surfaces) was based on the modified Triadan system (Floyd, 1991).

The animals were grouped according to the clinical periodontal condition: periodontitis when there was a periodontal pocket with a depth greater than 5 mm, with bleeding on probing and suppuration, clinically suggestive of a site with active lesions. Clinically healthy animals when there was no evidence of gingival alterations or periodontal pocket.

3.1. Collection of incisor dental biofilm

Dental biofilms from clinically healthy bovines were collected from the gingival sulcus of the medial labial surface of the incisor teeth (401 and 301), while biofilms of animals with periodontitis were collected from periodontal pockets of a depth greater than 5 mm and clinical signs that evidenced an active process, i.e., with bleeding on probing and suppuration (Dutra and Borsanelli, 2022).

The collection of dental biofilms was performed with a sterile Gracey curette after removing the supragingival biofilm with sterile gauze. The samples were kept in 250 ml of RNA later (Sigma-Aldrich, Dorset, United Kingdom), transported under refrigeration and stored at -80°C until processed (Borsanelli et al., 2018).

4. DNA extraction and amplicon sequencing

DNA was extracted from forest and pasture bulk soils using the DNeasy PowerSoil kit (MO BIO Laboratories, Inc.) following the manufacturer's protocol. For plant tissue, a cetyltrimethylammonium bromide (CTAB)-based method was applied (Doyle and Doyle, 1987), with adjustments to RNase A concentration according to forage type. Bovine oral microbiota was assessed by extracting DNA from dental biofilm samples using the GenElute Mammalian Genomic DNA Miniprep Kit (Sigma, St. Louis, USA) according to the manufacturer's instructions. The V4 region of the 16S rRNA gene was amplified using barcoded primers (Caporaso et al., 2012) with modifications to primer degeneracy (515F: GTGCCAGCMGCCGCGGTAA; 806R: GGACTACHVGGGTWTCTAAT) (Apprill et al., 2015). PCR products were purified, prepared into libraries, and sequenced on an Illumina MiSeq platform (paired-end, 251 bp reads). Peptide nucleic acid (PNA) PCR clamps were employed to suppress plastid and mitochondrial 16S sequences in plant tissue samples (Lundberg et al., 2013). Sequencing followed the Earth Microbiome Project protocol for 16S Illumina amplicons and was performed at the Argonne National Laboratory (USA). Raw sequences were demultiplexed in a Python environment based on primer barcodes. Subsequent processing was performed using the DADA2 pipeline (Callahan et al., 2019) with recommended parameters, including quality filtering for sequences longer than 250 bp. DADA2 identifies amplicon sequence variants (ASVs), which provide higher taxonomic resolution and reduce false positives compared to traditional OTU-based approaches (Callahan et al., 2016). Taxonomic assignment was carried out using the SILVA database (version 132) (Quast et al., 2012).

5. qPCR quantification of 16S rRNA and streptomycin biosynthesis gene (*strB1*)

Quantitative real-time PCR (qPCR) was employed to quantify the 16S rRNA and *strB1* genes in DNA samples from soil, forage, and cattle. The *strB1* gene is highly conserved among Streptomyces species and encodes a key component of the streptomycin biosynthetic pathway. Amplification of the 16S rRNA gene was performed using primers 1055yF (5'-ATGGYTGTCGTCAGCT-3') and 1392R (5'-ACGGGCGGTGTGTAC-3') (Ritalahti et al., 2006), whereas *strB1* was targeted using primers strB1F

(5’-ACTACGAGAGCCAGGAGCAGAT-3’) and strB1R (5’-TGACTCCGAGCTTGGTCAACT-3’) (Tanaka et al., 2009). DNA extracted from soils in forest and pasture systems was analyzed to assess gene abundance variations associated with forest-to-pasture conversion. Each qPCR reaction (20 µL) contained 1X SsoAdvanced Universal SYBR® Green Supermix (Bio-Rad Laboratories, Inc.), 300 nM of each primer, UltraPure DNase/RNase-free water (Invitrogen), and 2 µL of template DNA.

All reactions were performed in triplicate using a CFX96 Real-Time PCR system (Bio-Rad Laboratories, Inc.). No-template controls (PCR-grade water) were included to monitor contamination. Standard curves were generated by linear regression of quantification cycles against synthetic DNA standards (Integrated DNA Technologies, Iowa, USA). DNA standards were adjusted to 0.1 ng/µL and serially diluted (10-fold) to yield 3.65×10^1 to 3.65×10^8 gene copies per reaction. Thermal cycling conditions consisted of an initial step at 50 °C for 2 min, followed by 10 min at 95 °C. Amplification was performed over 40 cycles of denaturation at 95 °C for 15 s and annealing at 58 °C for 60 s for the 16S rRNA gene. For *strB1*, each cycle included annealing at 60 °C for 30 s and extension at 72 °C for 30 s. Fluorescence was recorded at the end of each annealing step.

References

Apprill, A., McNally, S., Parsons, R., Weber, L., 2015. Minor revision to V4 region SSU rRNA 806R gene primer greatly increases detection of SAR11 bacterioplankton. Aquatic Microbial Ecology 75, 129–137.

Borsanelli, A.C., Lappin, D.F., Viora, L., Bennett, D., Dutra, I.S., Brandt, B.W., Riggio, M.P., 2018. Microbiomes associated with bovine periodontitis and oral health. Vet Microbiol 218, 1–6.

Borsanelli, A.C., Viora, L., Lappin, D.F., Bennett, D., King, G., Dutra, I.S., Riggio, M.P., 2016. Periodontal lesions in slaughtered cattle in the west of Scotland. Veterinary Record 179.

Callahan, B., McMurdie, P., Holmes, S., 2019. DADA2 pipeline tutorial (1.8).

Callahan, B.J., McMurdie, P.J., Rosen, M.J., Han, A.W., Johnson, A.J.A., Holmes, S.P., 2016. DADA2: High-resolution sample inference from Illumina amplicon data. Nat Methods 13, 581–583.

Caporaso, J.G., Lauber, C.L., Walters, W.A., Berg-Lyons, D., Huntley, J., Fierer, N., Owens, S.M., Betley, J., Fraser, L., Bauer, M., Gormley, N., Gilbert, J.A., Smith, G., Knight, R., 2012. Ultra-high-throughput microbial community analysis on the Illumina HiSeq and MiSeq platforms. ISME J 6, 1621–1624. https://doi.org/10.1038/ismej.2012.8

dos Santos, H.G., Jacomine, P.K.T., Dos Anjos, L.H.C., De Oliveira, V.A., Lumbreras, J.F., Coelho, M.R., De Almeida, J.A., de Araujo Filho, J.C., de Oliveira, J.B., Cunha, T.J.F., 2018. Sistema brasileiro de classificação de solos. Brasília, DF: Embrapa, 2018.

Doyle, J.J., Doyle, J.L., 1987. A rapid DNA isolation procedure for small quantities of fresh leaf tissue.

Dutra, I.S., Borsanelli, A.C., 2022. Doenças periodontais. Saúde bucal de ruminantes: atlas para o reconhecimento das doenças periodontais 37–118.

Floyd, M.R., 1991. The modified Triadan system: nomenclature for veterinary dentistry. J Vet Dent 8, 18–19.

Lundberg, D.S., Yourstone, S., Mieczkowski, P., Jones, C.D., Dangl, J.L., 2013. Practical innovations for high-throughput amplicon sequencing. Nat Methods 10, 999–1002.

Miot, H.A., 2011. Sample size in clinical and experimental trials. J Vasc Bras 10, 275–278.

Quast, C., Pruesse, E., Yilmaz, P., Gerken, J., Schweer, T., Yarza, P., Peplies, J., Glöckner, F.O., 2012. The SILVA ribosomal RNA gene database project: improved data processing and web-based tools. Nucleic Acids Res 41, D590–D596.

Ritalahti, K.M., Amos, B.K., Sung, Y., Wu, Q., Koenigsberg, S.S., Löffler, F.E., 2006. Quantitative PCR targeting 16S rRNA and reductive dehalogenase genes simultaneously monitors multiple Dehalococcoides strains. Appl Environ Microbiol 72, 2765–2774.

Santos, R.D. dos, Lemos, R.C. de, Santos, H.G. dos, KER, J.C., Anjos, L.H.C. dos, Shimizu, S.H., 2005. Manual de descrição e coleta de solo no campo.

Tanaka, Y., Tokuyama, S., Ochi, K., 2009. Activation of secondary metabolite–biosynthetic gene clusters by generating rsmG mutations in Streptomyces griseus. J Antibiot (Tokyo) 62, 669–673.

Teixeira, P.C., Donagemma, G.K., Fontana, A., Teixeira, W.G., 2017. Manual de métodos de análise de solo. Rio de Janeiro, Embrapa. 573p.

WRB, I.W.G., 2015a. World reference base for soil resources 2014, update 2015: International soil classification system for naming soils and creating legends for soil maps. World Soil Resources Reports No. 106.

WRB, I.W.G., 2015b. World reference base for soil resources 2014, update 2015: International soil classification system for naming soils and creating legends for soil maps. World Soil Resources Reports No. 106.

Supplementary tables

Table S1. General information on properties classified as high and low herd-level prevalence of cattle periodontitis in the Brazilian Western Amazonia.

Sites	General information	Herd-level prevalence class
Bujari, AC[1]<br>9°49′22″S<br>67°56′51″W<br>196 m.a.s.l.	Farm 1: 5,900 ha of total area being 1,900 ha of pastures varying in conservation conditions; Herd of 5,000 cattle.<br>Pasture A: Newly formed pasture (< 7-year-old), predominance of Urochloa brizantha cv. MG5 and U. humidicola; Stagnic Luvisol (Luvissolo Háplico Pálico gleissólico, loamy/clayey texture, moderate A horizon) as predominant soil type.<br>Pasture B: 25-year-old pasture, U. decumbens predominantly; Stagnic Plinthosol (Plintossolo Argilúvico Alumínico gleissólico, loamy/clayey/very fine clayey texture, moderate A horizon).	Low (LSL)
Boca do Acre, AM 2<br>8°44′26″S<br>67°23′3″W<br>99 m.a.s.l.	Farm 2: 3,000 ha of total area, 1,000 ha of pastures; Herd of 1,000 cattle.<br>Pasture C: Newly formed pasture, predominance of Panicum maximum cv. Mombaça and U. humidicula; Xanthic Ferralsol (Latossolo Amarelo Alumínico típico, clayey/very fine clayey texture, moderate A horizon) as predominant soil type.<br>Pasture D: 30-year-old pasture, U. humidicola and U. brizantha cv. Marandu predominantly; Xanthic Ferralsol (Latossolo Amarelo Distrófico argissólico, loamy texture, moderate A horizon).	Low (LSL)
	Farm 3: 8,000 ha of total area, 5,000 ha with pastures; Herd of 6,000 cattle.<br>Pasture E: Newly formed pasture, predominance of U. brizantha cv. Xaraés and MG5; Pisoplinthic Ferralsol (Latossolo Amarelo Distrófico petroplíntico plintossólico, loamy/gravelly clayey texture, moderate A horizon) as predominant soil type.<br>Pasture F: 20-year-old pasture, U. brizantha cv. Marandu and U. humidicola. Plinthic Ferralsol (Latossolo Amarelo Distrófico plintossólico, clayey texture, moderate A horizon).	High (HSL)
Manicoré, AM<br>5°48′34″S<br>61°18′2″W<br>32 m.a.s.l.	Farm 4: 1,350 ha of total area, 400 ha with pastures; Herd of 300 cattle. Pasture G: Newly formed pasture, predominance of P. maximum cv. Mombaça; Xanthic Ferralsol (Latossolo Amarelo Distrófico argissólico, loamy texture, moderate A horizon) as predominant soil type.	High (HSL)
	Farm 5: 1,500 ha of total area, 550 ha with pastures; Herd of 800 cattle. Pasture H: Newly formed pasture, predominance of U. brizantha cv. MG5; Xanthic Ferralsol (Latossolo Amarelo Distrófico argissólico, loamy/clayey texture, moderate A horizon) as predominant soil type.	High (HSL)

[1] State of Acre; 2 State of Amazonas; * m.a.s.l. – meters above sea level; Soil classification followed the World Soil Reference (WRB, 2015b) and the Brazilian System of Soil Classification (DOS SANTOS et al., 2018).

Table S2. Characterization of soil physical and chemical variables of HSL and LSL pastures across Amazonian forest-to-pasture conversion landscapes; Mean values and standard deviation of 20 samples (per prevalence class).

Variable	Unit	High Prevalence Class (HSL)		Low Prevalence Class (LSL)	
CDW	g kg^{-1}	230.90	± 52.21	208.80	± 106.59
clay	g kg^{-1}	251.00	± 77.08	272.40	± 129.08
degree of flocculation	%	14.85	± 9.35	22.95	± 13.97
silt:clay ratio	-	0.46	± 0.25	2.00	± 1.22
silt	g $k^{-1}g^{-1}$	136.40	± 66.08	439.80	± 178.14
coarse sand	g kg^{-1}	283.25	± 225.10	38.40	± 17.80
Al	cmolc kg^{-1}	0.70	± 0.63	1.76	± 2.28
C	g kg^{-1}	15.87	± 2.98	16.81	± 3.67
Ca+Mg	cmolc kg	1.91	± 1.35	4.46	± 4.10
C:N ratio	-	10.20	± 1.11	8.15	± 1.14
H	cmolc kg^{-1}	5.50	± 1.75	6.59	± 2.12
H + Al	cmolc kg^{-1}	6.19	± 2.27	8.34	± 4.24
K	cmolc kg^{-1}	0.14	± 0.10	0.19	± 0.14
Al saturation (m%)	%	30.05	± 27.04	33.85	± 33.59
N	g kg^{-1}	1.57	± 0.30	2.06	± 0.49
Na	cmolc kg^{-1}	0.02	± 0.01	0.08	± 0.15
P	mg kg^{-1}	3.00	± 3.32	4.35	± 2.83
pHH_2O	-	5.10	± 0.71	5.18	± 0.55
sum of bases (SB)	cmolc kg^{-1}	2.07	± 1.41	4.72	± 4.18
CEC effective	cmolc kg^{-1}	8.26	± 1.51	13.07	± 4.07
base saturation (BS%)	%	26.20	± 18.18	34.35	± 24.97
Cu	mg kg^{-1}	0.57	± 0.43	1.20	± 0.31
Fe	mg kg^{-1}	125.20	± 75.16	162.70	± 112.45
Mn	mg kg^{-1}	13.62	± 14.95	116.81	± 109.65
Zn	mg kg^{-1}	0.80	± 0.59	2.68	± 2.03

Table S3. Characterization of bromatological variables in the forage of HSL and LSL pastures across Amazonian forest-to-pasture conversion landscapes; Mean values and standard deviation of 20 samples (per prevalence class).

Variable	Unit	HSL mean	HSL SD	LSL mean	LSL SD
Ether extract	%	1.75	± 0.55	1.40	± 0.36
Nitrogen-free extract	%	48.59	± 3.25	45.90	± 4.56
Acid detergent fiber	%	38.33	± 2.51	37.36	± 1.35
Neutral detergent fiber	%	66.26	± 4.29	64.27	± 2.92
Mineral matter	%	7.68	± 1.64	9.03	± 0.89
Total digestible nutrients	%	53.50	± 2.12	54.25	± 0.85
Total nitrogen (TN)	%	1.55	± 0.36	1.46	± 0.46
Non-protein nitrogen	% TN	26.19	± 15.36	21.62	± 1.63
Crude protein (CP)	%	9.65	± 2.24	9.14	± 2.90
Indigestible acid detergent fiber	%	1.13	± 0.58	1.16	± 0.22
Indigestible acid detergent fiber	% CP	12.40	± 6.20	13.54	± 3.77
Indigestible neutral detergent fiber	%	3.45	± 1.05	3.45	± 1.04
Indigestible neutral detergent fiber	% CP	38.75	± 15.93	38.03	± 7.06
Non-protein nitrogen (ProtA)	% total N	26.20	± 15.36	21.62	± 1.63
Soluble protein (ProtB1)	% total N	8.24	± 1.69	10.53	± 8.00
Insoluble protein (ProtB2, intermediate)	% total N	26.82	± 6.13	29.83	± 6.61
Insoluble protein (ProtB3, slow)	% total N	26.35	± 12.09	24.49	± 6.06
Indigestible protein (ProtC)	% total N	12.40	± 6.20	13.54	± 3.77
Rumen-degradable protein	% CP	31.25	± 9.13	31.50	± 14.66
Soluble protein	% CP	34.44	± 13.83	32.15	± 8.47
Ca	g kg-1	6.44	± 2.34	6.99	± 1.42
K	g kg-1	15.05	± 2.68	14.65	± 4.45
Mg	g kg-1	2.36	± 0.49	3.33	± 0.79
N	g kg-1	15.44	± 3.58	14.63	± 4.64
Na	%	0.08	± 0.01	0.07	± 0.00
P	g kg-1	0.89	± 0.11	1.27	± 0.25
Cu	mg kg-1	4.50	± 0.89	5.00	± 0.73
Fe	mg kg-1	206.50	± 141.59	229.25	± 74.68
Mn	mg kg-1	138.75	± 64.23	391.00	± 278.54
Zn	mg kg-1	12.50	± 1.54	20.00	± 7.64

Table S4. Rank of the 10 most important bacterial genera in soil, forage, and cattle as indicators of HSL and LSL systems of cattle periodontitis. Ranking is based on the IndValue and high statistical significance of each taxa.

Soil			Forage			Cattle		
**High Prevalence (HSL)**								
	**IndVal**	**p-value**		**IndVal**	**p-value**		**IndVal**	**p-value**
Leifsonia	0.727	<0.001	*Siporosoma*	0.680	<0,001	*Caviibacter*	0.674	<0.001
Occallatibacter	0.683	<0.001	*Hymenobacter*	0.602	<0,001	*Actinomyces*	0.668	<0.001
Dyella	0.652	<0.001	*Herbiconiux*	0.577	<0,001	*Campylobacter*	0.637	<0.001
Domibacillus	0.637	<0.001	*Herbaspirillum*	0.559	<0,001	*Porphyromonas*	0.632	<0.001
X1921.3	0.634	<0.001	*Xanthomonas*	0.552	<0,001	*Proteocatella*	0.618	<0.001
Kutzneria	0.632	<0.001	*Roeomonas*	0.544	<0,001	*Bergeyella*	0.613	<0.001
Sinomonas	0.626	<0.001	*Melittangium*	0.508	<0,001	*Bibersteinia*	0.613	<0.001
Rhodoplanes	0.620	<0.001	*Blastocatella*	0.450	<0,001	*Capnocytophaga*	0.613	<0.001
Roseiarcus	0.620	<0.001	*Psychroglaciecola*	0.424	<0,0015	*Filifactor*	0.558	<0.001
Acidothermus	0.619	<0.001	*Actinomycetospora*	0.423	<0,001	*Peptoanaerobacter*	0.550	<0.001
**Low Prevalence (LSL)**								
	**IndVal**	**p-value**		**IndVal**	**p-value**		**IndVal**	**p-value**
Luedemannella	0.805	<0.001	*Curtobacterium*	0.649	<0.001	*Ruminococcaceae NK4A214*	0.684	<0.001
Anaeromyxobacter	0.711	<0.001	*Aureimonas*	0.624	<0.001	*Corynebacterium*	0.667	<0.001
X1921.2	0.688	<0.001	*Novosphingobium*	0.578	<0.001	*Alysiella*	0.663	<0.001
Aquisfaera	0.685	<0.001	*Kineococcus*	0.501	<0.001	*Yersinia*	0.661	<0.001
Gemmata	0.671	<0.001	*Methylocella*	0.493	<0.001	*Streptococcus*	0.651	<0.001
ADurb.Bin063.1	0.657	<0.001	*Neorhizobium*	0.480	<0.001	*Moraxella*	0.632	<0.001
Rhodomicrobium	0.657	<0.001	*X1174.901.12*	0.431	<0.001	*Ruminococcus*	0.620	<0.001
Reyranella	0.648	<0.001	*Larkinella*	0.360	0,0064	*Haemophilus*	0.620	<0.001
Candidatus Xiphinematobacter	0.645	<0.001	*Enterococcus*	0.347	0.0101	*Christensenellaceae R.7*	0.614	<0.001
Ellin6067	0.645	<0.001	*Methylosinus*	0.320	0.0161	*Ruminococcaceae UCG.010*	0.614	<0.001

Table S5. Exact 16S V4 amplicon sequence variants (ASVs) detected in pasture soil, forage, and bovine subgingival biofilm samples.

ASV	Genus	Soil n/40	Forage n/35	Cattle n/27	Soil RA (%)	Forage RA (%)	Cattle RA (%)	HSL 3-way	LSL 3-way
ASV01	Acidibacter	5	1	1	0.023	0.004	0.027	No	No
ASV02	Acinetobacter	4	3	1	0.018	0.159	0.001	Yes	No
ASV03	Acinetobacter	1	1	1	<0.001	0.053	0.032	No	No
ASV04	Caulobacter	7	1	1	0.012	0.003	0.001	Yes	No
ASV05	Clostridium_sensu_stricto_1	6	1	1	0.013	0.003	0.006	No	No
ASV06	Escherichia/Shigella	6	1	24	0.038	0.026	0.957	Yes	No
ASV07	Janibacter	15	2	2	0.099	0.073	0.016	Yes	No
ASV08	Massilia	6	3	3	0.031	0.052	0.143	Yes	Yes
ASV09	Methylobacterium	13	27	6	0.018	1.979	0.034	Yes	Yes
ASV10	Pseudarthrobacter	18	3	1	0.081	0.019	0.015	No	Yes
ASV11	Pseudomonas	2	20	1	0.003	1.446	0.008	Yes	No
ASV12	Pseudomonas	2	27	1	0.002	4.823	0.002	No	No
ASV13	Ralstonia	4	6	1	0.010	0.098	0.030	Yes	No
ASV14	Romboutsia	10	1	5	0.018	0.002	0.095	No	Yes
ASV15	Sinomonas	11	1	1	0.077	0.002	0.009	No	No
ASV16	Staphylococcus	5	3	8	0.020	0.031	0.599	No	Yes
ASV17	Unclassified	20	3	5	0.106	0.110	0.068	Yes	Yes

RA, mean relative abundance (%) including nondetections; <0.001 denotes values below 0.001%. ASVs were matched by identical V4 nucleotide sequence after study filtering. HSL and LSL columns indicate whether an ASV was detected in all three compartments within that prevalence class. Nine ASVs met this criterion in HSL and six in LSL; three occurred in both classes, and five of the 17 pooled ASVs did not form a three-way overlap within either class. The 17 ASVs together accounted for mean relative abundances of 0.570% in soil, 8.884% in forage, and 2.042% in cattle. These descriptive counts were not compared statistically. Exact V4 identity does not establish strain identity or transmission.

Table S6. Topologies of co-occurrence networks based on abundance matrix of core microbiota from soil, forage, and cattle of HSL and LSL systems.

Topological measures	Core microbiota sub-communities					
	Soil		Forage		Cattle	
	LSL	HSL	LSL	HSL	LSL	HSL
Nodes [a]	66	58	19	25	22	34
Edges [b]	631	183	136	244	200	446
Average weighted degree [c]	14,226	4,741	5,172	7,247	6,826	9,324
Network diameter [d]	4	9	2	2	2	2
Graph density [e]	0.294	0.111	0.795	0.813	0.866	0.795
Modularity [f]	0.217	0.431	0.108	0.149	0.155	0.170
Number of communities [g]	4	5	2	2	3	3
Average clustering coefficient [h]	0.687	0.566	0.794	0.820	0.866	0.792

a Number of features with high significant biserial correlation coefficients ($p < 0.05$); **b** Number of connections obtained by pairwise correlations between nodes; **c** The average number of connections per node in the network, corrected according to the proportion of nodes and edges in the network; **d** The longest distance between nodes in the network, measured in number of edges; **e** Measures how much each node in a graph tend to cluster together; **f** The capability of the nodes to form a modular structure, that is, a structure with high density of nodes and clustered topology. **g** A community is defined as a group of nodes densely connected internally.

Supplementary figures

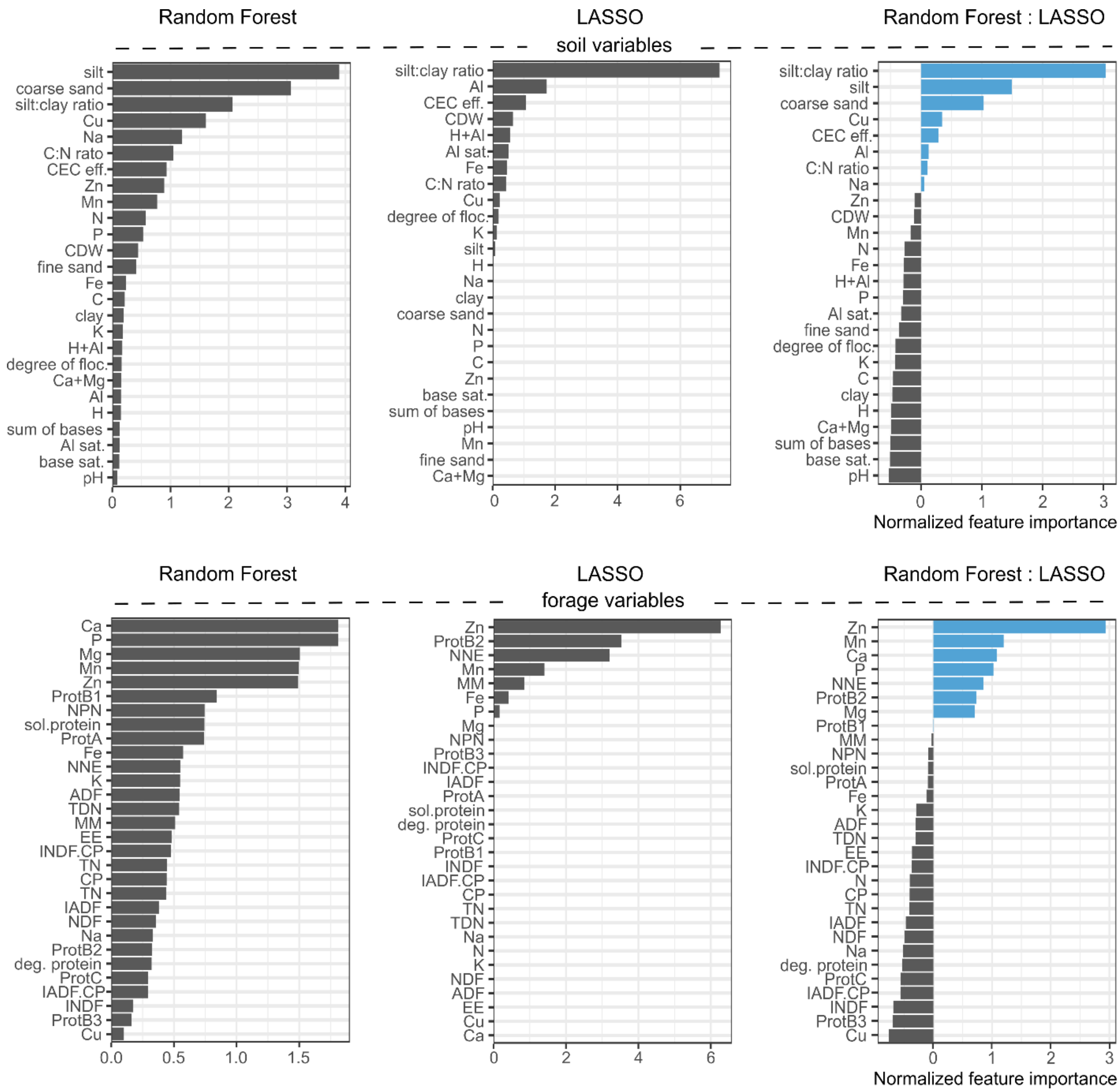

Figure S1. Variable selection of abiotic factor in soil (upper box) and forage (bottom box) from pastures of HSL and LSL systems. Individually is shown the selected variables by Random Forest and LASSO and the variables with the highest normalized feature importance retained by the integrated Random Forest : LASSO model.

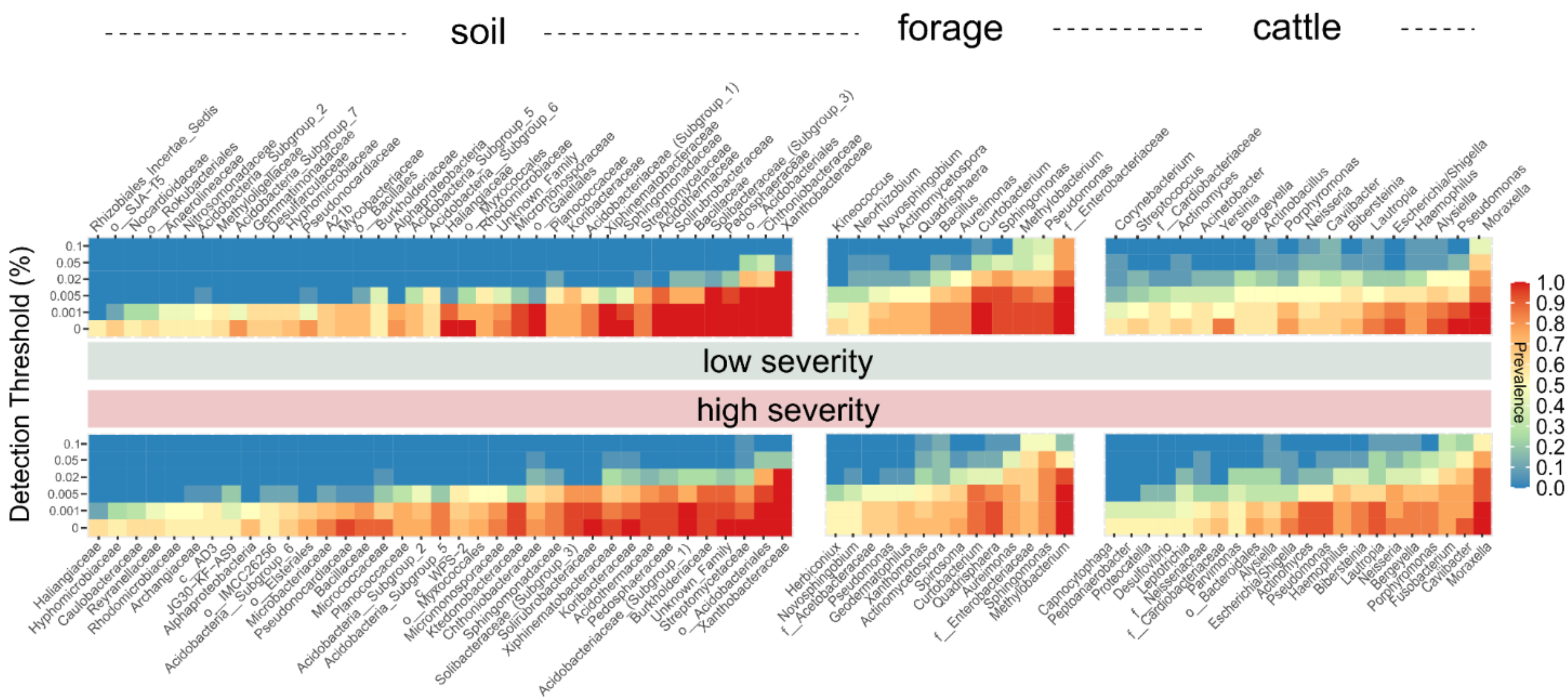

Figure S2. Heatmap with taxa ordered decreasingly according to their highest persistence and abundance in DNA samples from soil, forage, and cattle from HSL and LSL systems.

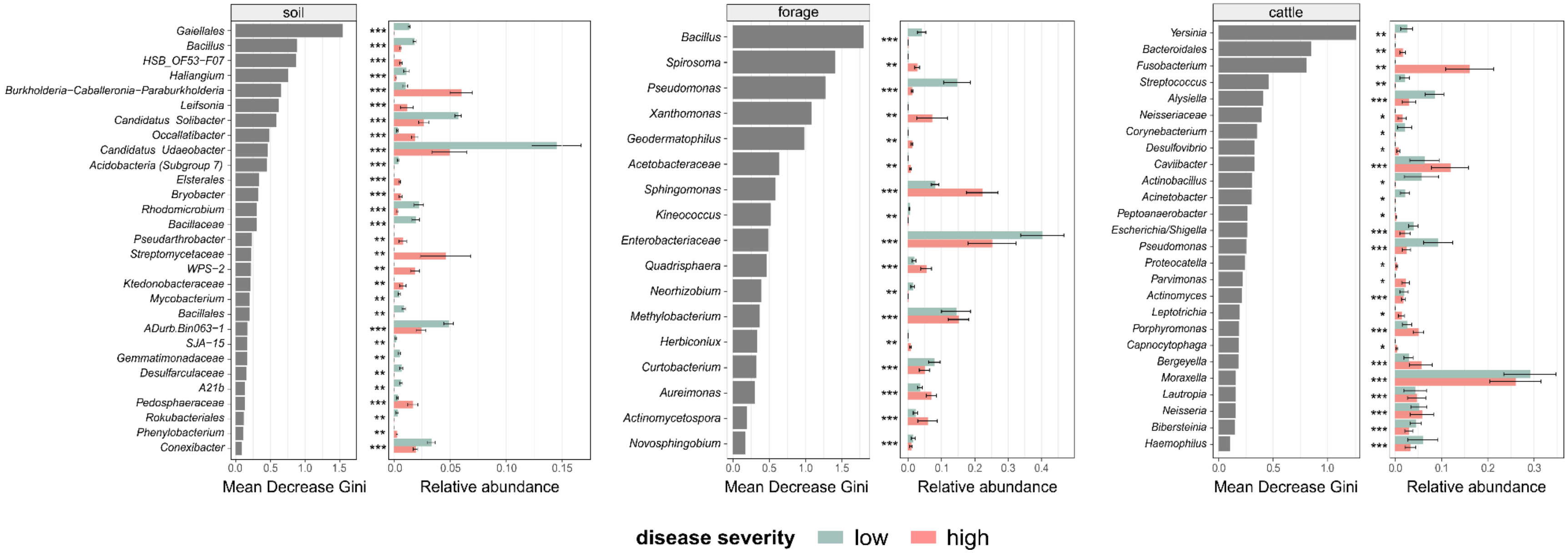

Figure S3. Random Forest feature-importance analysis of bacterial genera associated with HSL and LSL systems. Taxa were screened using Kruskal-Wallis tests with false-discovery-rate adjustment and ranked by mean decrease in Gini importance; the accompanying panels show relative abundance. Significance levels refer to FDR-adjusted p-values: (***) $p < 0.001$, (**) $p < 0.01$, (*) $p < 0.05$, n.s. not significant. Error bars indicate ± standard error (SE).

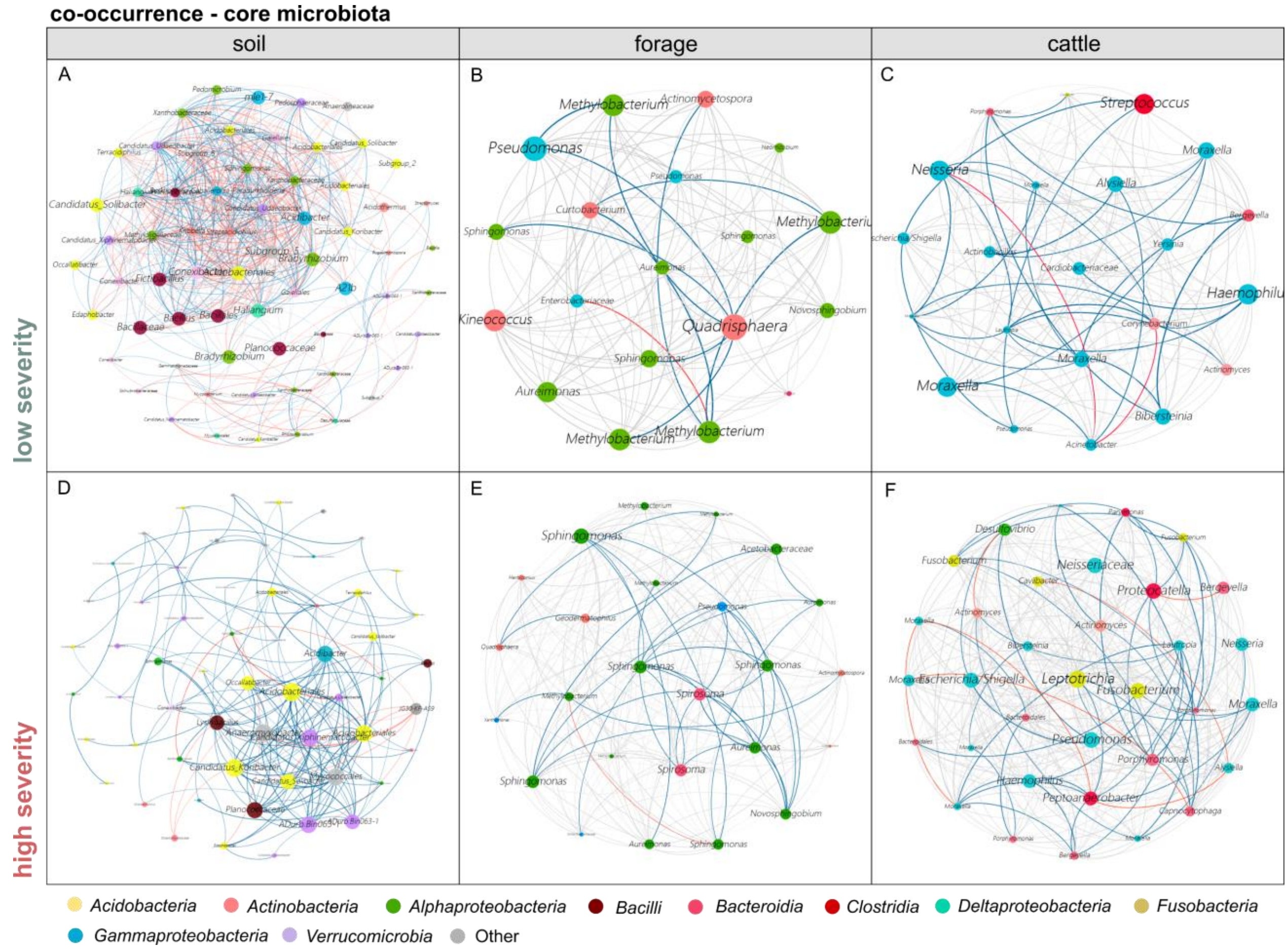

Figure S4. Co-occurrence network for the core community of soil, forage, and cattle from HSL and LSL systems. Blue lines refer to positive correlations and red to negative ones. Co-occurrence networks from the abundance matrix of soil, forage, and cattle prokaryotes from the HSL and LSL systems. Networks were constructed from taxa that showed at least one strong (Spearman; ≥ 0.70) and statistically significant ($p < 0.001$) correlation.

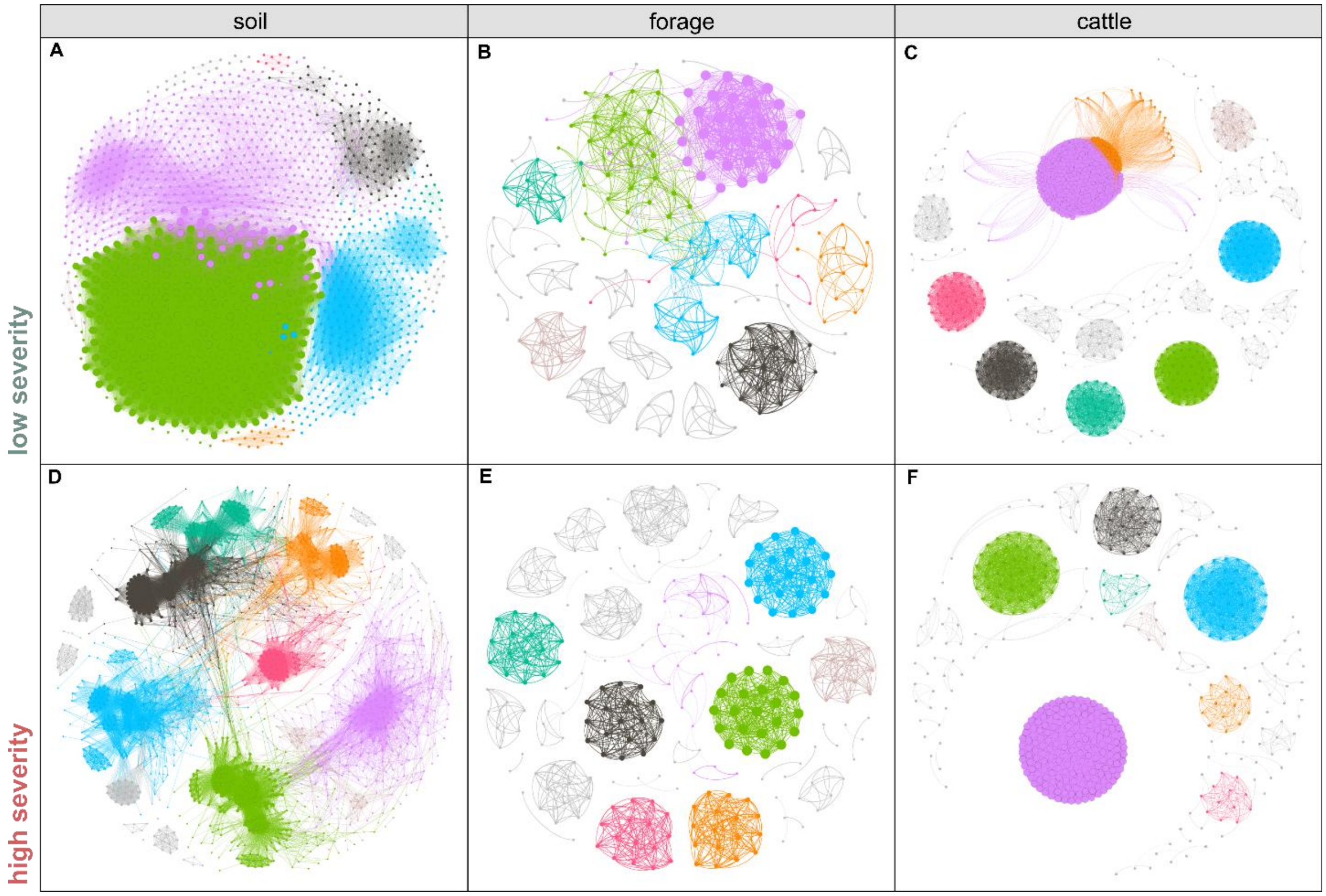

Figure S5. Co-occurrence networks from the abundance matrix of soil, forage, and cattle prokaryotes from the HSL and LSL systems. Networks were constructed from taxa that showed at least one strong (Spearman ≥ 0.70) and statistically significant ($p < 0.001$) correlation.

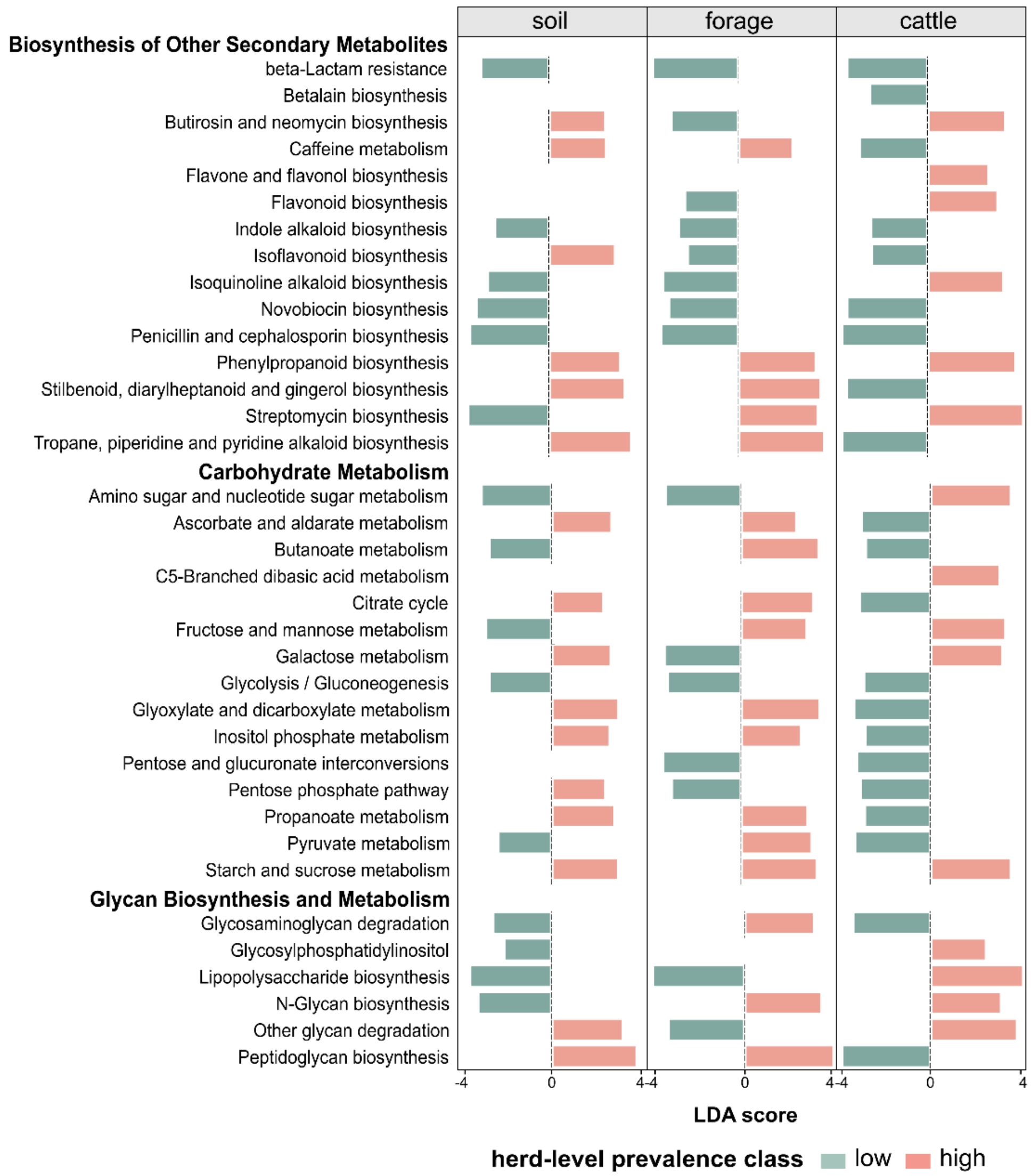

Figure S6. Differences in predicted metabolic potential across soil, forage, and cattle microbiomes from HSL and LSL systems. Tax4Fun-derived pathway profiles were evaluated using LEfSe with FDR-adjusted $p < 0.01$ and LDA score ≥ 3. These profiles represent predicted functional potential rather than measured microbial activity.
